# Isolation of Au-, Co-η^1^PCO and Cu-η^2^PCO complexes, conversion of an Ir–η^1^PCO complex into a dimetalladiphosphene, and an interaction-free PCO anion†
†Electronic supplementary information (ESI) available: Computational details and crystallographic data. CCDC 1404128–1404135, 1418457 and 1418458. For ESI and crystallographic data in CIF or other electronic format see DOI: 10.1039/c5sc04504e


**DOI:** 10.1039/c5sc04504e

**Published:** 2016-01-04

**Authors:** Liu Liu, David A. Ruiz, Fatme Dahcheh, Guy Bertrand, Riccardo Suter, Aaron M. Tondreau, Hansjörg Grützmacher

**Affiliations:** a UCSD-CNRS Joint Research Chemistry Laboratory (UMI 3555) , Department of Chemistry and Biochemistry , University of California San Diego , La Jolla , CA 92093-0343 , USA . Email: guybertrand@ucsd.edu; b Key Laboratory for Chemical Biology of Fujian Province , College of Chemistry and Chemical Engineering , Department of Chemistry , Xiamen University , Xiamen , 361005 , China; c Department of Chemistry and Applied Biosciences , ETH Zürich , Vladimir-Prelog-Weg 1 , 8093 Zürich , Switzerland . Email: hgruetzmacher@ethz.ch

## Abstract

Sodium phosphaethynolate reacts with [MCl(PDI)] (M = Co, Ir; PDI = pyridinediimine) to give metallaphosphaketenes, which in the case of iridium rearranges into a dimetalladiphosphene, *via* CO migration from phosphorus to the metal. Two different bonding modes of the PCO anion to CAAC-coinage metal complexes [CAAC: cyclic (alkyl)(amino)(carbene)] are reported, one featuring a strong Au–P bond and the other an η^2^ coordination to copper. The gold complex appears to be mostly unreactive whereas the copper complex readily reacts with various organic substrates. A completely free PCO anion was structurally characterized as the [Cu(L_a_)_2_]^+^ (OCP)^–^ salt. It results from the simple displacement of the PCO unit of the cationic (CAAC)Cu(PCO) complex by a second equivalent of CAAC.

## Abstract 2

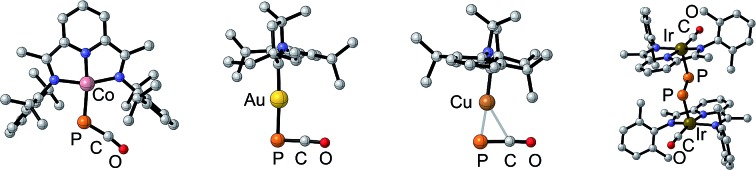
Sodium phosphaethynolate reacts with [MCl(PDI)] (M = Co, Ir; PDI = pyridinediimine) to give metallaphosphaketenes, which in the case of iridium rearranges into a dimetalladiphosphene.

## Introduction

**Fig. 1:**
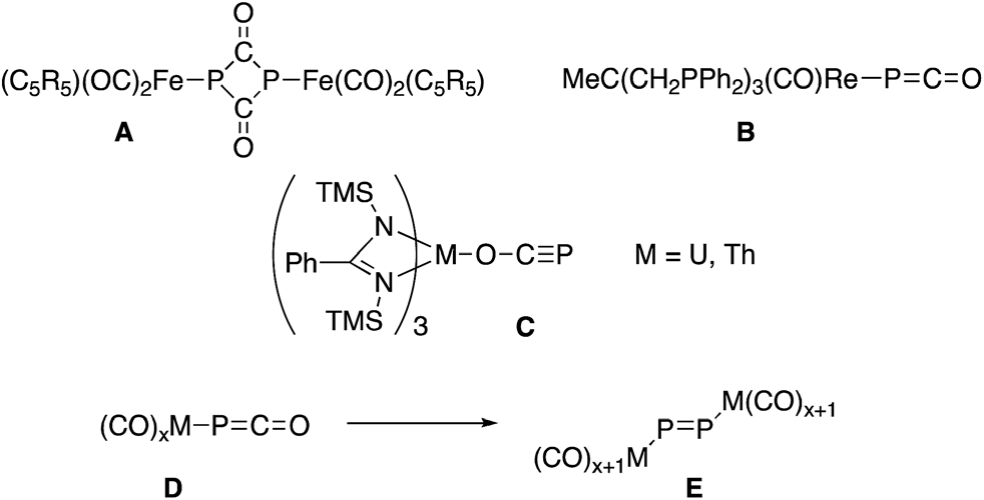
Previous experimental (**A–C**) and computational (**D–E**) works on phosphaethynolate with transition metals.^6^–^9^

Despite their early synthesis in 1992 by Becker, Westerhausen *et al.*,^1^ the reactivity of phosphaethynolate salts M^+^(PCO)^–^ remained unexplored until the recent development of simple syntheses which allow for the preparation of large quantities of pure material.^2^ These salts have been shown to undergo a variety of chemical transformations with organic substrates,^3^ including cycloaddition reactions leading to phosphorus containing heterocycles.^4^ In contrast, the strong reducing ability of PCO^–^ salts^5^ has hindered their exploration in transition metal chemistry. In most cases the reactions with metal complexes lead to decomposition products.^6^ The only exceptions are the formation of a P_2_(C=O)_2_ ring **A** by reacting Li(OCP) with (η^5^-C_5_R_5_)(CO)_2_FeBr,^7^ the isolation of [Re(PCO)(CO)_2_(triphos)] **B**,^6^ and uranium and thorium M(OCP)(amid)_3_ complexes **C**,^8^ in which the phosphaethynolate binds the metal *via* the oxygen center (Fig. 1). Noteworthy is an extensive computational study, showing that metallaphosphaketene complexes such as **D** may rearrange *via* CO migration into molecular metal phosphides **E**.^9^ The limited number of studies concerning the coordination chemistry of PCO^–^ is in marked contrast with the large number of reports dealing with the lighter analogue, namely the cyanate anion (NCO^–^).^10^

Herein we describe salt metathesis reactions leading to both unstable and stable terminal PCO transition metal complexes, featuring different coordination modes, and reactivity. We also report the experimental demonstration of the predicted conversion of **D** to **E**, and the displacement of the PCO unit of the copper complex by a CAAC, which leads to a PCO anion with no coordinating solvents or binding agents.

## Results and discussion

Because of the strongly reducing character of Na(OCP),^6^,^7^ we targeted complexes bearing pyridinediimine (PDI) ligands^11^ and cyclic (alkyl)(amino)carbenes (CAACs)^12^,^13^ which are known to efficiently stabilize metals in low oxidation states.^14^

**Fig. 2:**
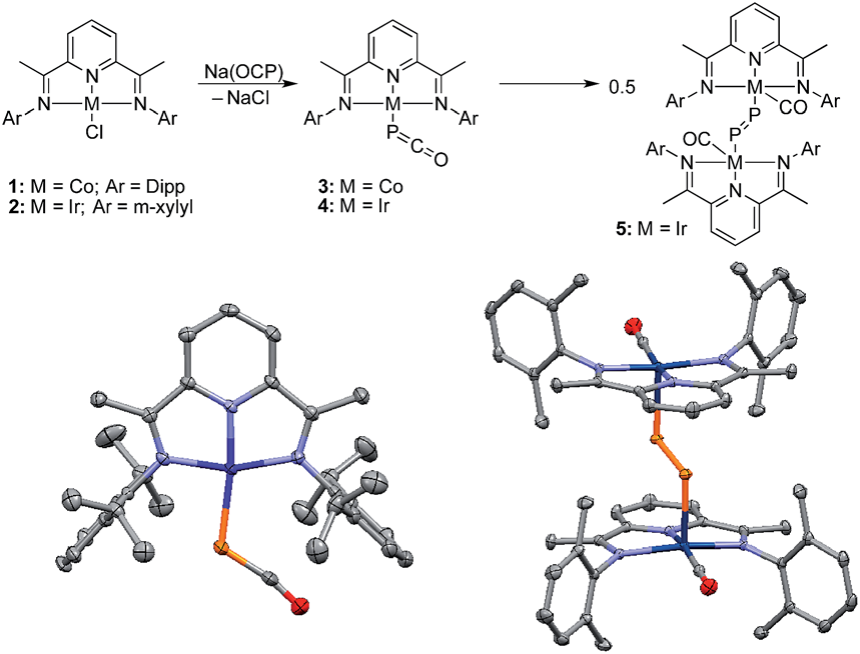
Synthesis of complexes **3** and **4**, and conversion of the latter into dimetalladiphosphene **5**, *via* CO migration. Solid-state structures of **3** (left) and **5** (right) (50% thermal ellipsoids are shown with hydrogen atoms and solvent molecules omitted for clarity); Dipp = 2,6-diisopropylphenyl, *m*-xylyl = 2,6-dimethylphenyl. Selected bond lengths [Å] and angles [°]: **3** P–Co 2.263(1), P–C 1.633(4), C–O 1.179(6); Co–P–C 116.2(1), P–C–O 169.9(4). **5** P–Ir 2.3886(6), P–P 2.021(1), C–Ir 1.862(2), C–O 1.153(3); Ir–P–P 103.76(3), C–Ir–P 92.787(6), Ir–C–O 177.8(2).

When the [CoCl(PDI^iPr^)] complex **1** was reacted with Na(OCP) in THF at –30 °C the color changed from pink to deep purple, and a single broad resonance in the ^31^P NMR spectrum at *δ* = –226 ppm [*vs. δ* = –392 ppm for Na(OCP)] indicated quantitative conversion. The metallaphosphaketene **3** was isolated in 61% yield and fully characterized. The IR spectrum showed the asymmetric stretching frequency of the phosphaketene unit at *ν*_asym_ = 1851 cm^–1^, intermediate between Na(OCP) (*ν*_asym_ = 1755 cm^–1^) and Ph_3_Sn–P=C=O (*ν*_asym_ = 1946 cm^–1^)2*d* indicating a cobalt phosphaketene structure, Co–P=C=O. This is confirmed by a single crystal X-ray diffraction analysis (Fig. 2). The P–C [1.633(4) Å] and C–O [1.179(6) Å] bond distances, the rather large Co–P–C angle [116.2(1)°] and long Co–C distance [3.325(4) Å] indicate a η^1^-coordination *via* the phosphorus atom of the OCP^–^ anion.^6^ The structural parameters confirm that neither the cobalt center nor the PDI ligand are reduced by OCP^–^.

The corresponding iridium complex [IrCl(PDI^Me^)] **2** with the less sterically encumbered PDI^Me^ ligand reacts with Na(OCP) at low temperatures to cleanly give product **4**. A ^31^P NMR resonance at *δ* = –316.7 ppm indicates a metallaphosphaketene (Ir–P=C=O) featuring a highly covalent metal phosphorus bond. Complex **4** could not be isolated. Keeping a THF solution at 20 °C for about 6 h leads cleanly to complex **5** (*δ*^31^P = +682 ppm; *λ*_max_ = 524.1 nm, 732.5 nm), which was isolated as red crystals. A single crystal X-ray structure analysis shows compound **5** to be a dimetalladiphosphene (Fig. 2).^15^ The P–P distance [2.021(1) Å] is short and in the typical range of diphosphenes, R–P=P–R. The iridium centers are bound to a redox-inactive PDI ligand with short C=N bonds [N1–C2 1.340(2) Å; N3–C8 1.335(2) Å] and a carbonyl ligand. The rearrangement of **4** into **5** is the experimental confirmation of the computationally predicted transformation of **D** to **E**.^9^ This transformation is also comparable to the conversion of an iridium azido complex, Ir–N_3_, to a transient terminal nitrido complex, Ir≡N, which could be spectroscopically characterized but also dimerizes to an Ir–N=N–Ir complex.^16^

**Fig. 3:**
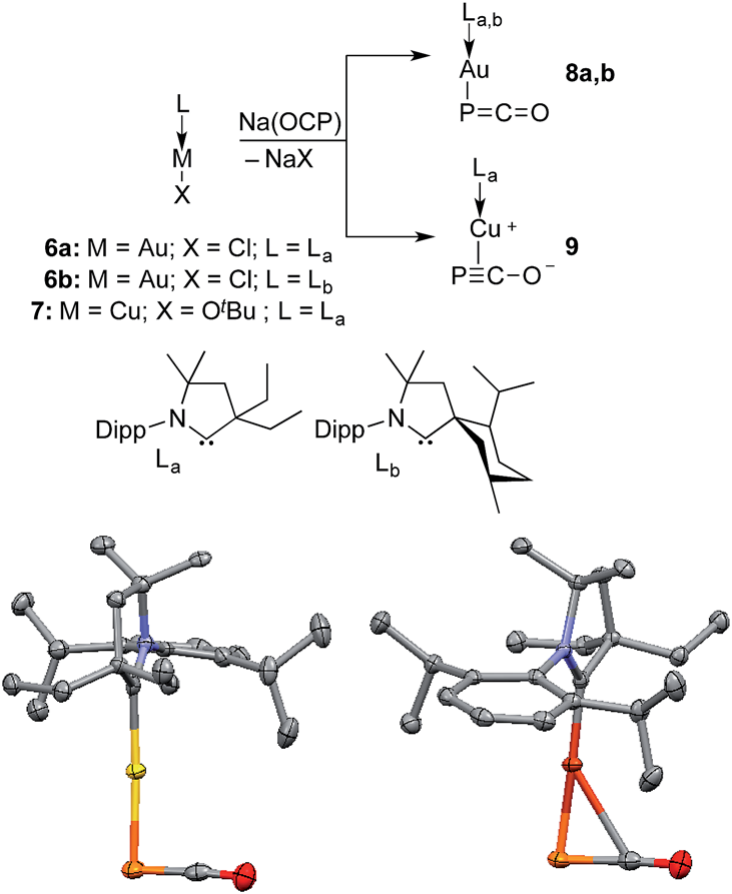
Synthesis and solid-state structures of **8a** (left) and **9** (right) (50% thermal ellipsoids are shown with hydrogen atoms omitted for clarity); Dipp = 2,6-diisopropylphenyl. Selected bond lengths [Å] and angles [°]: **8a** P–Au 2.354(1), P–C 1.640(3), C–O 1.176(4), Au–C 2.779(3); Au–P–C 86.2(1), P–C–O 176.5(3). **9** P–Cu 2.2244(5), P–C 1.636(2), C–O 1.184(2), Cu–C 2.501(2); Cu–P–C 79.15(5), P–C–O 176.4(1).

Monitoring by ^31^P NMR spectroscopy the reaction of gold and copper complexes **6a,b** and **7** with Na(OCP) in benzene showed in each case the formation of a single product giving a ^31^P NMR signal (**8a**: *δ* = –360; **8b**: –364; **9**: –387 ppm) slightly up-field shifted compared to that of Na(OCP) (*δ* = –392 ppm). Single crystals of **8a** and **9** were grown and subjected to X-ray diffraction studies (Fig. 3). Only very subtle structural differences between both complexes were observed. The P–C [**8a**: 1.640(3); **9**: 1.636(2) Å] and C–O bond lengths [**8a**: 1.176(4); **9**: 1.184(2) Å] are similar, and the M–P–C angle is slightly more acute for the copper complex **9** [**8a**: 86.2(1), **9**: 79.15(5)°].

**Fig. 4:**
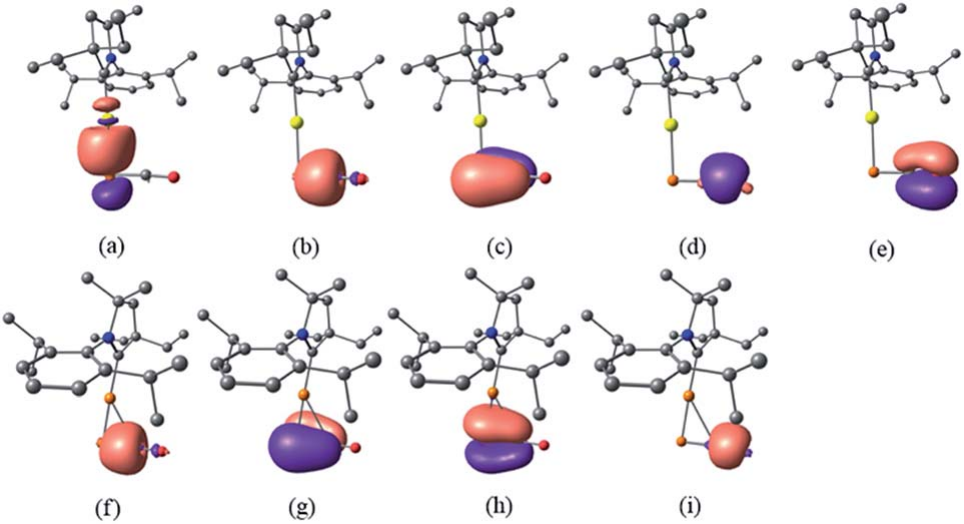
NBOs corresponding to M(PCO) fragments of **8a** and **9** (isovalue = 0.05). NBOs of **8a**: (a) Au–P σ; (b) P–C σ; (c) P–C π; (d) C–O σ; (e) C–O π. NBOs of **9**: (f) P–C σ; (g) P–C π; (h) P–C π; (i) C–O σ.

Despite the similarities of the solid state structures, natural bond orbital (NBO) analysis at the M06/6-311++G(2d,p)+SDD//M06/6-31+G(d)+LANL2DZ(+f) level of theory shows significant differences between the electronic structures of **8a** and **9**. The NBO charges of Au and PCO in **8a** are +0.39 and –0.56 a.u., respectively, whereas those of Cu and PCO in **9** are +0.58 and –0.68 a.u., suggesting that the PCO anion in **9** is more ionic and “free” than that in **8a**. This is in agreement with the different ^31^P NMR chemical shifts of **8a** (*δ* = –360 ppm) and **9** (*δ* = –387 ppm). The NBOs corresponding to the M(PCO) (M = Au or Cu) fragments are quite different as shown in Fig. 4. The phosphorus center of **8a** forms three bonds (Au–P σ, P–C σ and P–C π) (Fig. 4a–c). In contrast, for **9** no Cu–P σ bond could be located. Instead, the phosphorus center of **9** forms one P–C σ bond and two P–C π bonds (Fig. 4f–h). Moreover, there are two bonds between the C and O atoms (C–O σ and C–O π) in **8a** (Fig. 4d and e), while only one C–O σ bond in **9** (Fig. 4i). These computational results suggest that the coordination modes of PCO with gold and copper are η^1^ and η^2^, respectively.

**Fig. 5:**
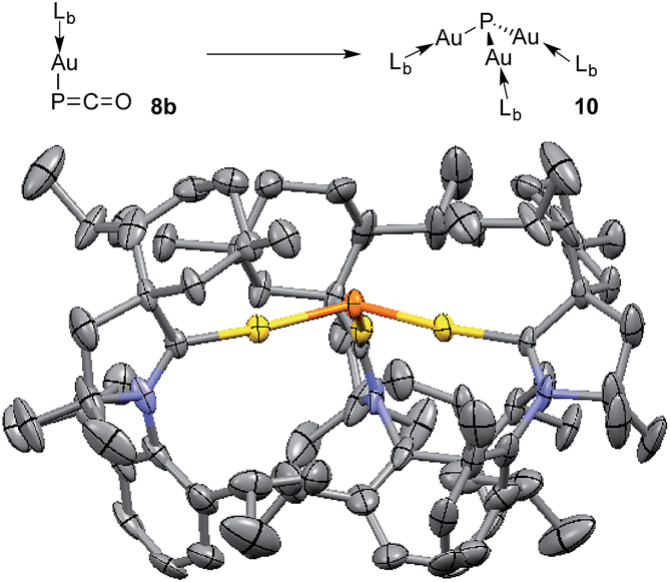
Conversion of **8b** to (L_b_Au)_3_P **10**, and solid state structure of **10** (50% thermal ellipsoids are shown with hydrogen atoms omitted for clarity).

The different bonding modes in **8** and **9** lead to a difference in reactivity. In solution **9** decomposes after a few hours giving a complex mixture, whereas **8b** rearranges into complex **10** over the course of a week when left standing in THF at room temperature (Fig. 5). The trinuclear nature of **10** [(L_b_Au)_3_P], as determined by an X-ray diffraction study, is reminiscent of the rearrangement product of Ph_3_Sn(PCO), namely (Ph_3_Sn)_3_P.2d,^17^ Three gold atoms surround a single P atom, leading to a phosphine supported solely by metals. The ^31^P NMR spectrum displays a signal at *δ* = –200 ppm, which is considerably high-field shifted compared to alkyl and aryl phosphines. The electron rich nature as well as the steric crowding around the P center could make **10** an interesting redox active ligand for transition metals.^18^

**Fig. 6:**
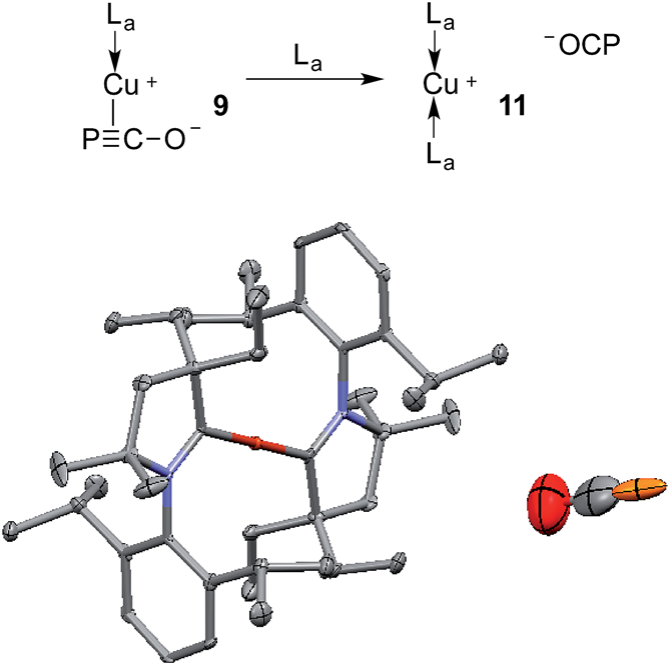
Solid-state structure of **11** showing the free PCO anion. 50% thermal ellipsoids are shown and hydrogen atoms are omitted for clarity.

The gold complexes **8a,b** are rather inert and do not react with heavier group 14 element halides to give R_3_E-PCO derivatives. Equally, no reaction with *N*,*N*′-dicyclohexylcarbodiimide or with carbene L_a_ are observed. On the contrary, the copper salt **9** does react with these reagents similarly to Na(OCP) (see the ESI† for details).2b,d Remarkably, **9** reacts with carbene L_a_ to afford the cationic bis(CAAC)Cu complex **11**, in which the PCO fragment is the anionic counterpart. The ^31^P NMR signal appears at *δ* = –400 ppm, which is more downfield shifted than Na(OCP) (*δ* = –392 ppm), implying that PCO^–^ is less coordinated. The IR spectrum showed the asymmetric stretching frequency of the PCO unit at *ν*_asym_ = 1791 cm^–1^, suggesting a more cumulenic nature than in the two crystalline forms of Na(OCP) (*ν*_asym_ = 1780 or 1755 cm^–1^).^6^,19a,b Lastly, although a disorder precludes accurate determination of the geometric parameters, the X-ray diffraction study revealed that the PCO anion has no close contacts with the cationic part of the complex (Fig. 6). This is the first time that the PCO anion has been structurally observed without any binding agents or coordinating solvents.

**Fig. 7:**
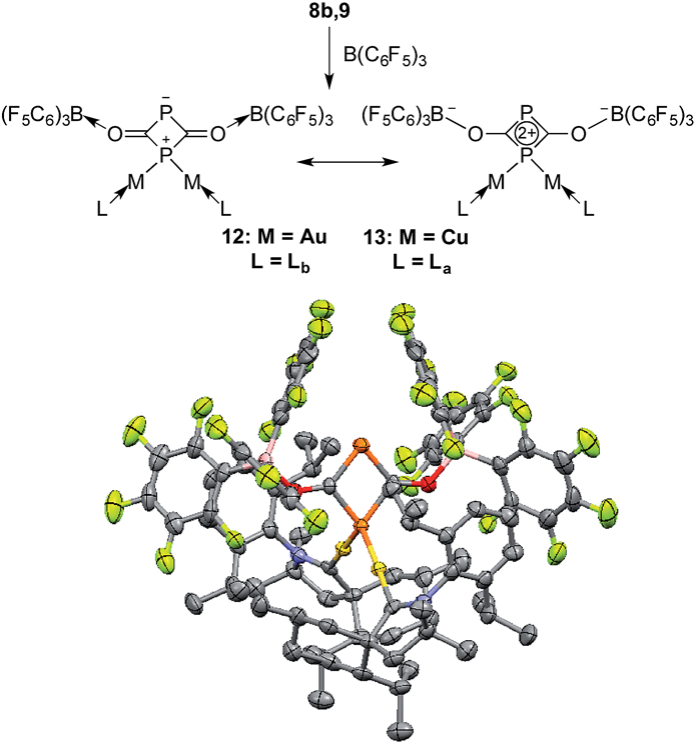
Reaction of **8b**,**9** with B(C_6_F_5_)_3_ and two resonance structures of **12** and **13**; solid state-structure of the gold complex **12** (50% thermal ellipsoids are shown with hydrogen atoms omitted for clarity).

On the basis of DFT calculations, the HOMOs of **8a** and **9** are mainly localized on the PCO fragments (see ESI†) and the oxygen atoms carry the largest negative charge (–0.56 a.u. in **8a** and –0.59 a.u. in **9**). Thus, we were curious to see if the terminal oxygen atom could react with a Lewis acidic borane. Indeed, adding one equivalent of B(C_6_F_5_)_3_ to either **8b** or **9** led to the same type of heterocycle **12** and **13**, respectively (Fig. 7). In the case of gold, the corresponding product immediately crystallized out and became insoluble in the tested solvents. However, for copper the product was soluble and the ^31^P NMR spectrum showed two broad peaks at *δ* = 261 and *δ* = 137 ppm. Single crystal X-ray diffraction studies of **12** and **13** revealed a four-membered P_2_C_2_ heterocycle that arose from borane coordination to oxygen followed by a dimerization and lastly a migration of a LM fragment from one phosphorus center to the other. The four-membered P_2_C_2_ heterocycles have a planar geometry and, as expected, the bond lengths of the PCO fragments become elongated compared to **8b** and **9** as a result of the delocalization over the ring. This small ring represents a novel bonding mode for the rapidly growing field of molecular polyphosphorus clusters and cages.^20^

## Conclusions

PCO complexes with electron rich metal centers such as copper, gold and also cobalt can be prepared and are relatively stable. On the other hand, the iridium phosphaketene **4** rapidly rearranges *via* CO migration to give a genuine dimetalladiphosphene. The copper complex features an η^2^ coordination mode, which leads to an active PCO fragment that can undergo further reactions. A free PCO anion, resulting from simple displacement of the PCO unit with a carbene, was also isolated. For both the copper and gold complexes, borane coordination to the oxygen of the OCP unit induces a [2 + 2] cycloaddition into a P_2_C_2_ heterocycle. These results demonstrate that the uncharted chemistry of transition metal PCO complexes is rich and the formation of new metal phosphides and the mechanisms leading to them merit further exploration.

## Experimental section

### General considerations

All air- and moisture-sensitive manipulations were carried out using standard vacuum line Schlenk techniques or an MBraun dry-box under argon. THF was distilled over sodium benzophenone-ketyl before use. THF-*d*_8_, CD_2_Cl_2_, and C_6_D_6_ were purchased from Cambridge Isotope Laboratories and dried over 4 Å molecular sieves. (^iPr^PDI)CoCl **1**,^21^ (^Me^PDI)IrCl **2**,11*d* L_a,b_AuCl **6a,b**^22^ and L_a_CuOtBu **7**^23^ were synthesized according to literature procedures. ^1^H, ^13^C, ^11^B, ^19^F, and ^31^P NMR spectra were recorded on a Varian VX 500, Bruker 300, Bruker 500 and Jeol 500 spectrometer at 25 °C. All ^1^H and ^13^C NMR chemical shifts are reported relative to SiMe_4_ using the ^1^H (residual) and ^13^C chemical shifts of the solvent as a secondary standard. NMR multiplicities are abbreviated as follows: s = singlet, d = doublet, t = triplet, sept = septet, m = multiplet, br = broad signal. Chemical shifts are given in ppm and coupling constants *J* are given in Hz. Peak widths at half heights (in Hz) are given for broad signals. Infrared spectra were collected on a Perkin-Elmer-Spectrum 2000 FT-IR-Raman and Bruker ALPHA FT-IR spectrometer. Elemental analyses were performed at the Mikrolabor of ETH Zürich. Single crystals suitable for X-ray diffraction were coated with polyisobutylene oil in a dry-box, transferred to a nylon loop and then transferred to the goniometer of a Bruker X8 APEX2 diffractometer equipped with a molybdenum X-ray tube (*λ* = 0.71073 Å) or on a Bruker Apex II-CCD detector using Mo-Kα radiation (*λ* = 0.71073 Å) or Cu-Kα radiation (*λ* = 1.54178 Å). The data were processed using the Bruker SAINT+ program and corrected for absorption using SADABS. The structures were solved using direct methods (SHELXS) completed by Fourier synthesis and refined by full-matrix least-squares procedures. Mass spectra were performed at the UC San Diego Mass Spectrometry Laboratory. Melting points were measured with an electrothermal MEL-TEMP apparatus.

#### Preparation of (^iPr^PDI)Co(PCO) **3**

In the glove box, a 20 mL scintillation vial was charged with 0.200 g (0.347 mmol) of (^iPr^PDI)CoCl **1** and 10 mL of THF. The solution was cooled to –35 °C and Na(OCP) (0.130 g, 0.355 mmol) was added portion-wise over the course of 5 minutes, eliciting a color change from pink to dark purple. The reaction was placed in the freezer at –35 °C for one hour then filtered through Celite. The solution was concentrated, layered with hexane and placed at –35 °C. This gave 0.152 g (48%) of a purple crystalline solid identified as [(^iPr^PDI)Co(PCO)] **3**. The mother liquor was placed back in the freezer to obtain another 42 mg (13%) of product. X-Ray quality crystals were grown from the second fraction. Analysis for C_34_H_43_CoN_3_OP, 599.64 g mol^–1^, calc.: C, 68.10; H, 7.23; N, 7.01 found: C, 66.05; H, 7.35; N, 6.85. IR (powder): *ν* PCO = 1851 cm^–1^. ^1^H NMR (C_6_D_6_, 500 MHz): *δ* = 9.66 (t, *J* = 7.6 Hz, 1H, CH_Pyr_), 7.47 (t, *J* = 7.7 Hz, 2H, CH_arom_), 7.35 (d, *J* = 7.7 Hz, 4H, CH_arom_), 7.05 (d, *J* = 7.6 Hz, 2H, CH_Pyr_), 3.32 (sept, *J* = 6.7 Hz, 4H, ^i^Pr(CH)), 1.13 (dd, *J* = 2.4 Hz, *J* = 6.7 Hz, 24H, ^i^Pr(CH_3_)), –0.19 (s, 6H, CH–CH_3_); ^13^C NMR (C_6_D_6_, 125 MHz): *δ* = 181.2 (d, *J*_PC_ = 98.8 Hz, P*C*O), 168.1, 153.4, 150.5, 140.0, 125.1, 124.0, 116.4, 28.6 (Ar-*C*H_3_), 24.0 (Ar-*C*H_3_), 23.3, 21.8; ^31^P NMR (C_6_D_6_, 202 MHz): *δ* = –225.8 ppm (lb = 634 Hz).

#### Preparation of [(^Me^PDI)IrCO]_2_(μ-P_2_) **5**

A 20 mL Schlenk flask was charged with 0.100 g (0.167 mmol) of (MePDI)IrCl **2** and 5 mL of THF and cooled in a dry-ice/acetone bath. A solution of Na(OCP) (0.060 g, 0.170 mmol) in THF (3 mL) was syringed into the stirring iridium solution, immediately causing a color change to dark purple. The reaction was warmed to room temperature, whereupon the color changed to deep pink, and stirred for an additional hour. The reaction was then filtered through Celite and then concentrated to roughly 3 mL. Storing at –35 °C overnight produced a solid that was collected on a glass frit and dried under reduced pressure, yielding 0.076 g (74% yield) of red crystalline solid **5**. X-Ray quality crystals were grown from the slow evaporation of the mother liquor at room temperature overnight. NMR analysis was performed in CD_2_Cl_2_ due to the poor solubility of **5** in ethereal or aromatic solvents, but the compound slowly decomposed (if left overnight) in methylene chloride. Analysis for C_52_H_55_Ir_2_N_6_O_2_P_2_, calcd: C 50.27, H 4.46, N 6.76; found: C 49.96, H 4.63, N 6.31. IR (powder): *ν* CO = 1967 cm^–1^. ^1^H NMR (CD_2_Cl_2_, 500 MHz): *δ* = 8.12 (d, *J* = 7.8 Hz, 2H, *m*-Py), 7.26 (br s, 1H, *p*-Py), 7.07–6.93 (m, 6H), 2.3 (br s, 6H, CN–*CH*_*3*_), 1.63 (s, 6H, Ar-*CH*_*3*_), 1.43 (s, 6H, Ar-*CH*_*3*_); ^13^C NMR (CD_2_Cl_2_, 125 MHz): *δ* = 187.50 (*C*O), 152.66, 149.21, 143.12, 131.89, 130.36, 128.28 (Ar-*C*H), 127.96 (Ar-*C*H), 126.02, (Ar-*C*H), 123.97 (*m*-Py-*C*H), 116.16 (*m*-Py-*C*H), 20.66 (Ar-*C*H_3_), 18.31 (Ar-*C*H_3_), 15.51 (CN–*C*H_3_); ^31^P NMR (CD_2_Cl_2_, 202 MHz): *δ* = 683.2.

#### Preparation of (CAAC)Au(PCO) complex **8a**

A mixture of (CAAC)AuCl **6a** (1.0 g, 1.83 mmol) and [Na(PCO)(dioxane)_2.5_] (0.55 g, 1.83 mmol) was cooled to –78 °C before THF (10 mL) was added. The mixture was stirred for 15 minutes and then warmed to room temperature. After 30 min, the solvent was removed under vacuum and the resulting brown solid was extracted with 15 mL of benzene. After removing the solvent, **8a** was obtained as a light yellow solid (0.73 g, yield: 70%). Colorless single crystals of **8a** were obtained by vapor diffusion of pentane into a saturated benzene solution of **8a** in the dark. IR (C_6_H_6_): *ν* PCO = 1887 cm^–1^. M.P. = 193 °C (dec.). ^1^H NMR (C_6_D_6_, 500 MHz): *δ* = 7.13 (t, 1H, *J* = 7.3 Hz), 7.00 (d, 2H, *J* = 7.3 Hz), 2.72 (sept, 2H, *J* = 6.6 Hz), 1.63 (m, 4H), 1.54 (d, 6H, *J* = 6.6 Hz), 1.42 (s, 2H), 1.08 (d, 6H, *J* = 6.6 Hz), 0.85 (m, 12H); ^13^C {^1^H} NMR (C_6_D_6_, 125 MHz): *δ* = 253.5 (C_carbene_ d, *J*_PC_ = 36.2 Hz), 183.0 (C=O d, *J*_PC_ = 100.4 Hz), 146.1, 135.3, 130.9, 126.0, 81.0, 63.0, 43.0, 32.4, 20.1, 29.5, 27.7, 23.7, 10.3; ^31^P {^1^H} NMR (C_6_D_6_, 121 MHz) *δ* = –359.5. HRMS was attempted but a peak corresponding to M^+^ could not be located, probably due to the weak P metal bond.

#### Preparation of (CAAC)Au(PCO) complex **8b**

A mixture of (CAAC)AuCl **6b** (1.0 g, 1.63 mmol) and [Na(PCO) (dioxane)_2.5_] (0.49 g, 1.63 mmol) was cooled to –78 °C before THF (10 mL) was added. The mixture was stirred for 15 minutes and then warmed to room temperature. After 30 min, the solvent was removed under vacuum and the resulting brown solid was extracted with 15 mL of benzene. After removing the solvent, **8b** was obtained as a light yellow solid (0.70 g, yield: 67%). Colorless single crystals of **8b** were obtained by vapor diffusion of (TMS)_2_O into a saturated benzene solution of **8b** in the dark. IR (C_6_H_6_): *ν* PCO = 1889 cm^–1^. M.P. = 221 °C (dec.). ^1^H NMR (C_6_D_6_, 500 MHz): *δ* = 7.14 (br, 1H), 7.00 (br, 2H), 3.17 (br, 2H), 2.79 (sept, 2H, *J* = 6.8 Hz), 2.05 (m, 1H), 1.95 (m, 1H), 1.88 (d, 1H, *J* = 12.4 Hz), 1.82 (m, 1H), 1.69 (d, 2H, *J* = 12.4 Hz), 1.58 (d, 3H, *J* = 6.8 Hz), 1.52 (d, 3H, *J* = 6.8 Hz), 1.26 (d, 3H, *J* = 6.8 Hz), 1.09 (m, 9H), 0.93 (m, 9H), 0.87 (d, 3H, *J* = 6.8 Hz); ^13^C {^1^H} NMR (C_6_D_6_, 125 MHz): *δ* = 253.5 (C_carbene_ d, *J*_PC_ = 37.1 Hz), 182.5 (C=O d, *J*_PC_ = 101.1 Hz), 146.2, 145.8, 136.1, 130.6, 129.2, 125.8, 77.6, 65.4, 53.4, 52.0, 50.0, 36.4, 31.3, 30.1, 29.8, 28.8, 28.0, 27.3, 25.6, 23.8, 23.7, 23.6, 20.7; ^31^P {^1^H} NMR (C_6_D_6_, 121 MHz) *δ* = –364.2. HRMS was attempted but a peak corresponding to M^+^ could not be located, probably due to the weak P metal bond.

#### Preparation of (CAAC)Cu(PCO) complex **9**

A mixture of (CAAC)CuOtBu **7** (50 mg, 0.11 mmol) and [Na(OCP) (dioxane)_2.5_] (35 mg, 0.11 mmol) was stirred for 10 minutes in 3 mL of benzene at room temperature. The solvent was removed under vacuum and the resulting brown solid was washed with 10 mL of pentane. After drying under vacuum, **9** was obtained as a light yellow solid (30 mg, yield: 62%). Colorless single crystals of **9** were obtained were obtained by vapor diffusion of (TMS)_2_O into a saturated toluene solution of 9 at –40 °C. IR (C_6_H_6_): *ν* PCO = 1849 cm^–1^. M.P. = 173 °C (dec.). ^1^H NMR (C_6_D_6_, 500 MHz): *δ* = 7.12 (t, 1H, *J* = 7.7 Hz), 7.00 (d, 2H, *J* = 7.7 Hz), 2.75 (sept, 2H, *J* = 6.8 Hz), 1.68 (m, 4H), 1.43 (d, 6H, *J* = 6.8 Hz), 1.38 (s, 2H), 1.08 (d, 6H, *J* = 6.8 Hz), 0.93 (m, 6H), 0.85 (s, 6H); ^13^C {^1^H} NMR (C_6_D_6_, 125 MHz): *δ* = 251.8 (C_carbene_), 175.2 (C=O d, *J*_PC_ = 97.8 Hz), 145.9, 135.5, 130.7, 125.6, 81.2, 63.1, 43.1, 31.9, 29.9, 29.4, 27.9, 23.1, 10.4; ^31^P {^1^H} NMR (C_6_D_6_, 121 MHz) *δ* = –387.4. HRMS was attempted but a peak corresponding to M^+^ could not be located, probably due to the weak P metal bond.

#### Preparation of (L_b_Au)_3_P **10**

Complex **8b** (50 mg, 0.078 mmol) was left standing in 2 mL of THF for 1 week under visible light. White crystals of **10** were generated and washed with 5 mL of pentane (10 mg, yield: 22%). M. P. = 293 °C (dec.). ^1^H NMR (CD_2_Cl_2_, 500 MHz): *δ* = 7.39 (t, 3H, *J* = 8.4 Hz), 7.18 (m, 6H), 2.72 (m, 12H), 2.28 (m, 3H), 1.88 (m, 9H), 1.78 (m, 3H), 1.67 (m, 3H), 1.34 (m, 9H), 1.24 (m, 36H), 1.13 (d, 9H, *J* = 6.6 Hz), 0.98 (m, 18H), 0.90 (d, 9H, *J* = 6.6 Hz), 0.82 (d, 9H, *J* = 6.6 Hz); ^13^C {^1^H} NMR (CD_2_Cl_2_, 125 MHz): *δ* = 260.3 (C_carbene_ d, *J*_PC_ = 84 Hz), 146.3, 145.7, 135.3130.2, 125.5, 125.2, 79.0, 78.9, 66.5, 66.4, 52.9, 50.5, 36.5, 30.5, 30.4, 30.0, 29.9, 29.5, 29.0, 28.9, 28.5, 25.2, 25.1, 24.5, 23.3, 23.2, 21.3; ^31^P {^1^H} NMR (CD_2_Cl_2_, 121 MHz) *δ* = –200.2. HRMS: *m*/*z* calculated for [C_81_H_130_N_3_Au_3_P]^+^ (M + H)^+^ 1766.8999; found 1766.8980.

#### Preparation of bis(CAAC)Cu^+^PCO^–^ complex **11**

A mixture of **9** (20 mg, 0.046 mmol) and carbene L_a_ (15 mg, 0.049 mmol) was stirred for 2 min in benzene (0.5 mL). The suspension was filtered and the colorless powder was washed with benzene (1 mL), yielding **11** (30 mg, 86% yield). Single crystals were obtained by slow evaporation of a saturated benzene solution of **11**. IR (solid, KBr): PCO *ν* = 1791 cm^–1^. M.P. = 158 °C (dec.). ^1^H NMR (C_6_D_6_, 500 MHz): *δ* = 7.08 (t, 2H, *J* = 7.8 Hz), 6.97 (d, 4H, *J* = 7.8 Hz), 2.70 (sept, 4H, *J* = 6.8 Hz), 1.54 (m, 8H), 1.42 (d, 12H, *J* = 6.8 Hz), 1.32 (s, 4H), 1.05 (d, 12H, *J* = 6.8 Hz), 0.84 (t, 12H, *J* = 7.2 Hz), 0.81 (s, 12H); ^13^C {^1^H} NMR (C_6_D_6_, 125 MHz): *δ* = 253.3 (C_carbene_), 145.8, 135.3, 130.7, 125.5, 80.8, 63.0, 43.6, 31.6, 30.0, 29.4, 28.0, 23.1, 10.2; ^31^P {^1^H} NMR (C_6_D_6_, 121 MHz) *δ* = –399.5. HRMS: *m*/*z* calculated for [C_44_H_70_CuN_2_]^+^ 689.4835; found 689.4847.

#### Preparation of four-membered heterocycle **12**

A mixture of **8b** (50 mg, 0.078 mmol) and B(C_6_F_5_)_3_ (40 mg, 0.078 mmol) was stirred in 2 mL of benzene for 5 minutes. The resulting yellow suspension was filtered and the yellow residue was washed with benzene (0.5 mL), then dried under vacuum, yielding 61 mg (64%) of a bright-yellow powder. Yellow single crystals of **12** were obtained in the filtrate in less than 1 min. M.P. = 230 °C (dec.).

#### Preparation of four-membered heterocycle **13**

A mixture of **9** (50 mg, 0.12 mmol) and B(C_6_F_5_)_3_ (59 mg, 0.12 mmol) was stirred in 1 mL of toluene for 5 minutes. The solvent was removed under reduced pressure and the residue was washed with pentane (2 mL), yielding 68 mg (62%) of a light-yellow powder. Colorless single crystals were obtained by vapor diffusion of (TMS)_2_O into a saturated toluene solution of **13**. M.P. = 160 °C (dec.). ^1^H NMR (C_6_D_6_, 500 MHz): *δ* = 7.08 (t, 2H, *J* = 8.1 Hz), 6.93 (d, 4H, *J* = 8.1 Hz), 2.59 (sept, 4H, *J* = 6.6 Hz), 1.40 (m, 8H), 1.31 (m, 4H), 1.12 (d, 12H, *J* = 6.6 Hz), 1.08 (d, 12H, *J* = 6.6 Hz), 1.01 (m, 12H), 0.85 (m, 12H); ^13^C {^1^H} NMR (C_6_D_6_, 125 MHz): *δ* = 248.8 (C_carbene_ br), 149.5 (br d, *J*_FC_ = 238 Hz), 149.0 (br d, *J*_FC_ = 247 Hz), 145.5, 138.1 (br d, *J*_FC_ = 247 Hz), 135.1, 131.2, 125.8, 82.5, 62.8, 43.0, 31.5, 29.7, 29.2, 27.7, 22.7, 9.9; ^31^P {^1^H} NMR (C_6_D_6_, 121 MHz) *δ* = 260.6 (br), 136.2 (br). HRMS was attempted but a peak corresponding to M^+^ could not be located, probably due to the weak P metal bond.

## Supplementary Material

Supplementary information

Crystal structure data
