# Supplementary material for: Isolation of Au-, Co-η1PCO and Cu-η2PCO complexes, conversion of an Ir–η1PCO complex into a dimetalladiphosphene, and an interaction-free PCO anion
Source: Chem Sci. 2016 Jan 4;7(3):2335–41. doi: 10.1039/c5sc04504e (PMC6003603; doi:10.1039/c5sc04504e)
Supplement: Supplementary file 1 [file SC-007-C5SC04504E-s001.pdf]

Electronic Supplementary Material (ESI) for Chemical Science.
This journal is © The Royal Society of Chemistry 2016

Electronic Supplementary Information

Isolation of Au-, Co-η^1PCO and Cu-η^2PCO complexes, conversion of an Ir-η^1PCO complex into a dimetalladiphosphene, and an interaction-free PCO anion

Liu Liu, David A. Ruiz, Fatme Dahcheh, Guy Bertrand,* Riccardo Suter, Aaron M. Tondreau, and Hansjörg Grützmacher*

Index

1. Experimental Details---2-21
2. Crystallographic Data---22-25
3. Computational Details---26-32

General Information

All air- and moisture-sensitive manipulations were carried out using standard vacuum line Schlenk techniques or an MBraun dry-box under argon. THF was distilled over sodium benzophenone-ketyl before use. THF-d_8, CD_2Cl_2, and C_6D_6 were purchased from Cambridge Isotope Laboratories and dried over 4 Å molecular sieves. ^{1}H, ^{13}C, ^{11}B, ^{19}F, and ^{31}P NMR spectra were recorded on a Varian VX 500, Bruker 300, Bruker 500 and Jeol 500 spectrometer at 25 °C. All ^{1}H and ^{13}C NMR chemical shifts are reported relative to $SiMe_4$ using the ^{1}H (residual) and ^{13}C chemical shifts of the solvent as a secondary standard. NMR multiplicities are abbreviated as follows: *s* = singlet, *d* = doublet, *t* = triplet, *sept* = septet, *m* = multiplet, *br* = broad signal. Chemical shifts are given in ppm and coupling constants *J* are given in Hz. Peak widths at half heights (in Hz) are given for broad signals. Infrared spectra were collected on a Perkin-Elmer-Spectrum 2000 FT-IR-Raman spectrometer. Elemental analyses were performed at the Mikrolabor of ETH Zürich. Single crystals suitable for X-ray diffraction were coated with polyisobutylene oil in a dry-box, transferred to a nylon loop and then transferred to the goniometer of a Bruker X8 APEX2 diffractometer equipped with a molybdenum X-ray tube (λ = 0.71073 Å) or on a Bruker Apex II-CCD detector using Mo-Kα radiation (λ = 0.71073 Å) or Cu-Kα radiation (λ = 1.54178 Å). The data were processed using the Bruker SAINT+ program and corrected for absorption using SADABS. The structures were solved using direct methods (SHELXS) completed by Fourier synthesis and refined by full-matrix least-squares procedures. Mass spectra were performed at the UC San Diego Mass Spectrometry Laboratory. Melting points were measured with an electrothermal MEL-TEMP apparatus

Synthesis and characterization

Preparation of (^{iPr}PDI)Co(PCO) 3: In the glove box, a 20 mL scintillation vial was charged with 0.200 g (0.347 mmol) of (^{iPr}PDI)CoCl **1** and 10 mL of THF. The solution was cooled to -35 °C and Na(OCP) (0.130 g, 0.355 mmol) was added portion-wise over the course of 5 minutes, eliciting a color change from pink to dark purple. The reaction was placed in the freezer at -35 °C for one hour then filtered through Celite. The solution was concentrated, layered with hexane and placed at -35 °C. This gave 0.152 g (48%) of a purple crystalline solid identified as [(^{iPr}PDI)Co(PCO)] **3**. The mother liquor was placed back in the freezer to obtain another 42 mg (13%) of product. X-Ray quality crystals were grown from the second fraction. Analysis for $C_{34}H_{43}CoN_3OP$, 599.64 g/mol, calc: C, 68.10; H, 7.23; N, 7.01 Found: C, 66.05; H, 7.35; N, 6.85. IR (powder): ν PCO = 1851 cm^{-1}. ^{1}H NMR (C_6D_6, 500 MHz): δ = 9.66 (t, *J* = 7.6 Hz, 1 H, CH_{Pyr}), 7.47 (t, *J* = 7.7 Hz, 2 H, CH_{arom}), 7.35 (d, *J* = 7.7 Hz, 4 H, CH_{arom}), 7.05 (d, *J* = 7.6 Hz, 2 H, CH_{Pyr}), 3.32 (sept, *J* = 6.7 Hz, 4 H, ^{i}Pr(CH)), 1.13 (dd, *J* = 2.4 Hz, *J* = 6.7 Hz, 24 H, ^{i}Pr(CH_3)), -0.19 (s, 6 H, CH-CH_3); ^{13}C NMR (C_6D_6, 125 MHz): δ = 181.2 (d, J_{PC} = 98.8 Hz, P*C*O), 168.1, 153.4, 150.5, 140.0, 125.1, 124.0, 116.4, 28.6 (Ar-*C*H_3), 24.0 (Ar-*C*H_3), 23.3, 21.8; ^{31}P NMR (C_6D_6, 202 MHz): δ = -225.8 ppm (lb = 634 Hz).

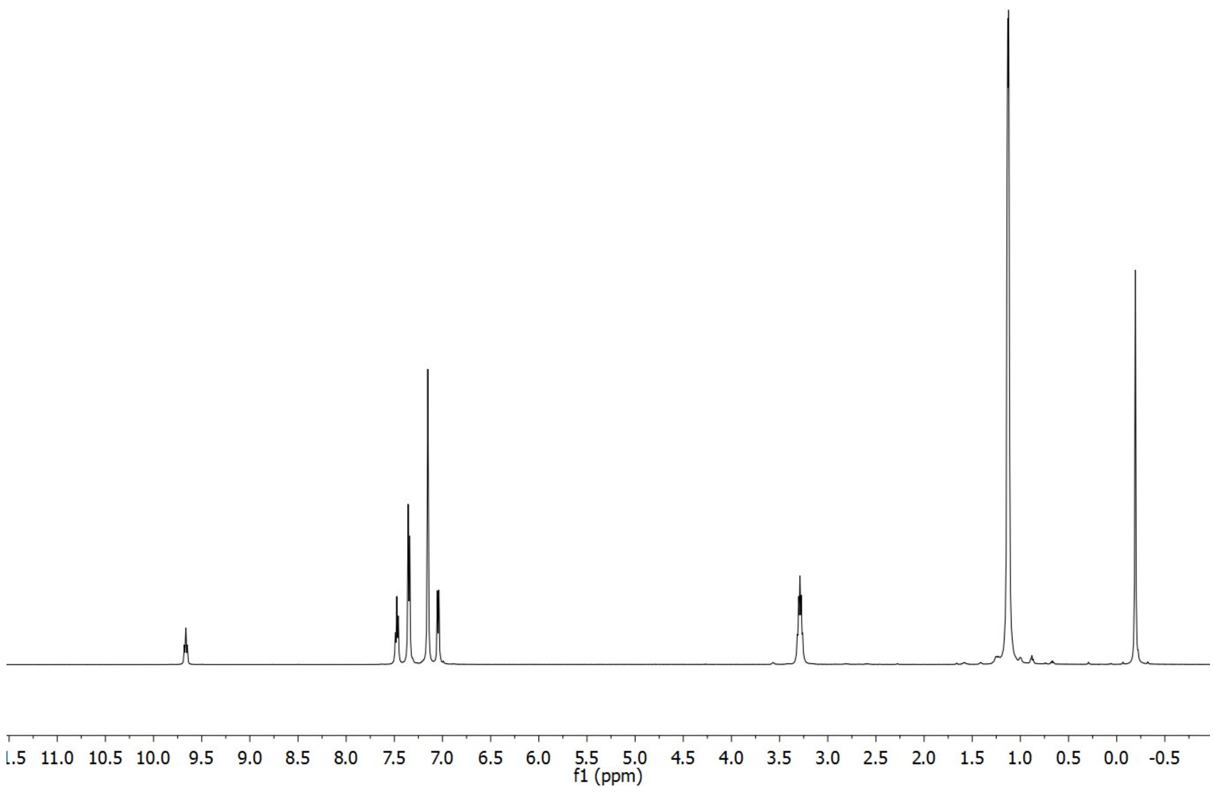
1.5 11.0 10.5 10.0 9.5 9.0 8.5 8.0 7.5 7.0 6.5 6.0 5.5 5.0 4.5 4.0 3.5 3.0 2.5 2.0 1.5 1.0 0.5 0.0 -0.5
f1 (ppm)

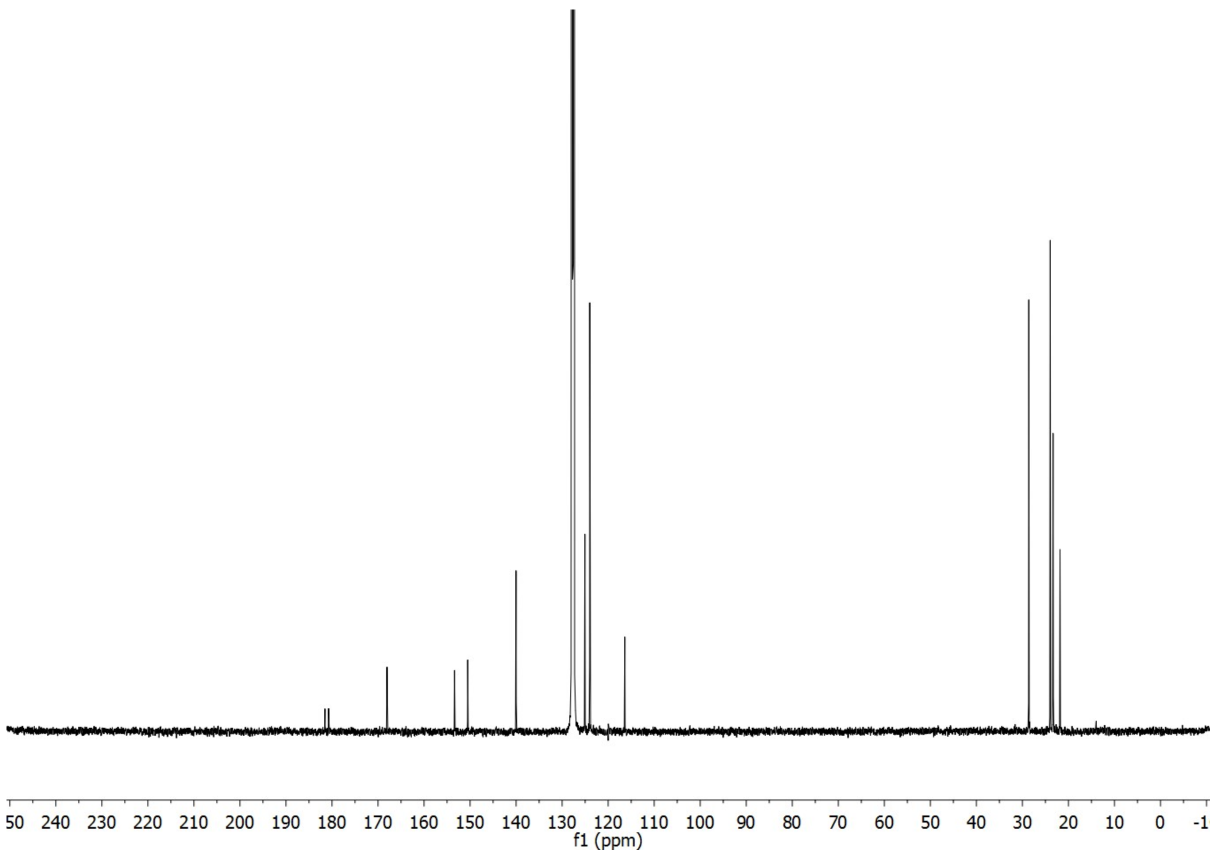
50 240 230 220 210 200 190 180 170 160 150 140 130 120 110 100 90 80 70 60 50 40 30 20 10 0 -1
f1 (ppm)

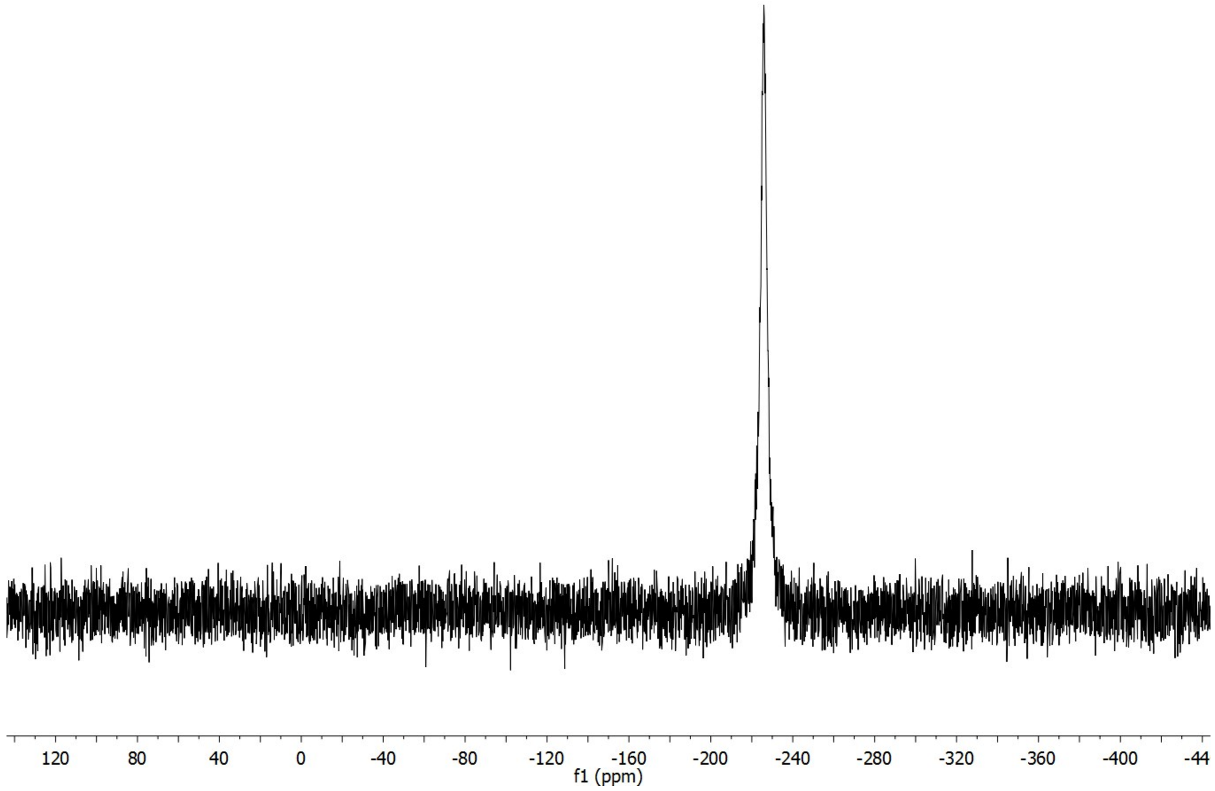

Preparation of [(^{Me}PDI)IrCO]$_2$(μ-P$_2$) 5: A 20 mL Schlenk flask was charged with 0.100 g (0.167 mmol) of **2** and 5 mL of THF and cooled in a dry-ice/acetone bath. A solution of Na(OCP) (0.060 g, 0.170 mmol) in THF (3 mL) was syringed into the stirring iridium solution, immediately causing a color change to dark purple. The reaction was warmed to room temperature, whereupon the color changed to deep pink, and stirred for an additional hour. The reaction was then filtered through Celite and then concentrated to roughly 3 mL. Storing at -35 °C overnight produced a solid that was collected on a glass frit and dried under reduced pressure, yielding 0.076 g (74% yield) of red crystalline solid **5**. X-Ray quality crystals were grown from the slow evaporation of the mother liquor at room temperature overnight. NMR analysis was performed in CD_2Cl_2 due to the poor solubility of **5** in ethereal or aromatic solvents, but the compound slowly decomposed (if left overnight) in methylene chloride. Analysis for $C_{52}H_{55}Ir_2N_6O_2P_2$, Calcd: C 50.27, H 4.46, N 6.76; Found: C 49.96, H 4.63, N 6.31. IR (powder): ν CO = 1967 cm^{-1}, IR (solid, KBr): ν CO = 1953 cm^{-1}. ^{1}H NMR (CD_2Cl_2, 500 MHz): δ = 8.12 (d, *J* = 7.8 Hz, 2 H, *m*-Py), 7.26 (br s, 1 H, *p*-Py), 7.07-6.93 (m, 6 H), 2.3 (br s, 6 H, CN-*CH$_3$*), 1.63 (s, 6 H, Ar-*CH$_3$*), 1.43 (s, 6 H, Ar-*CH$_3$*); ^{13}C NMR (CD_2Cl_2, 125 MHz): δ = 187.50 (*C*O), 152.66, 149.21, 143.12, 131.89, 130.36, 128.28 (Ar-*C*H), 127.96 (Ar-*C*H), 126.02, (Ar-*C*H), 123.97 (*m*-Py-*C*H), 116.16 (*m*-Py-*C*H), 20.66 (Ar-*C*H$_3$), 18.31 (Ar-*C*H$_3$), 15.51 (CN-*C*H$_3$); ^{31}P NMR (CD_2Cl_2, 202 MHz): δ = 683.2. λ_{max} [nm] : 524.1 nm, 732.5 nm.

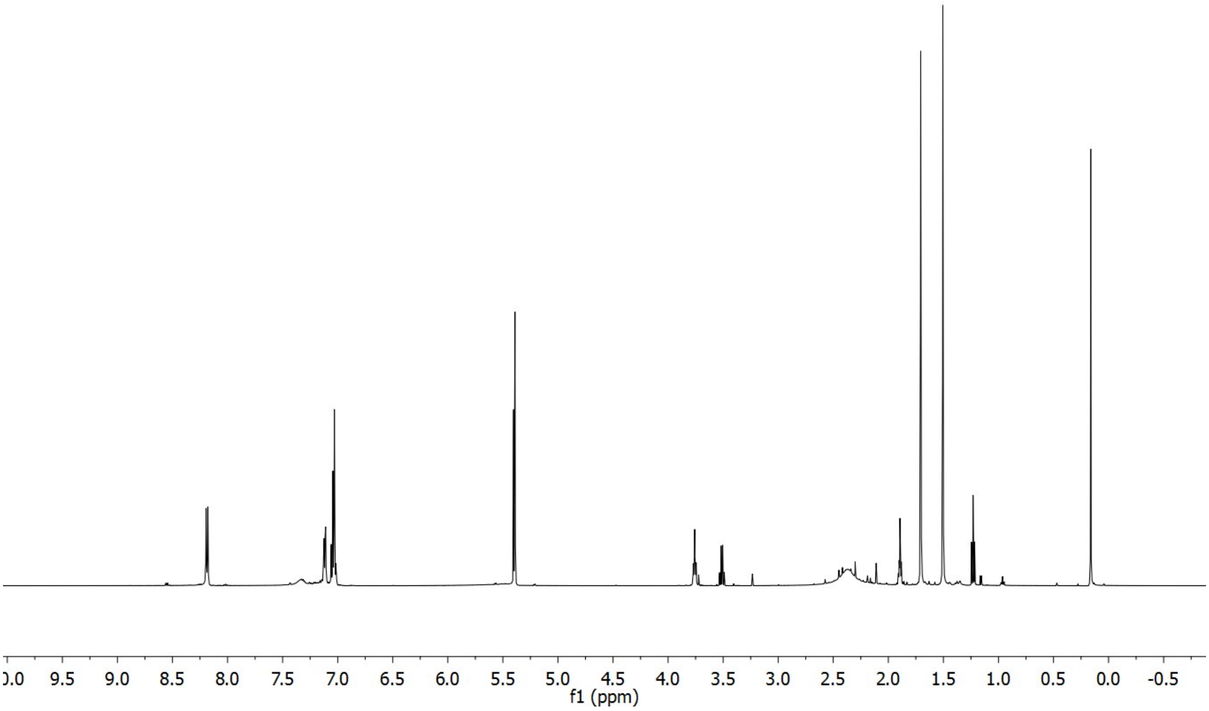
9.5 9.0 8.5 8.0 7.5 7.0 6.5 6.0 5.5 5.0 4.5 4.0 3.5 3.0 2.5 2.0 1.5 1.0 0.5 0.0 -0.5
f1 (ppm)

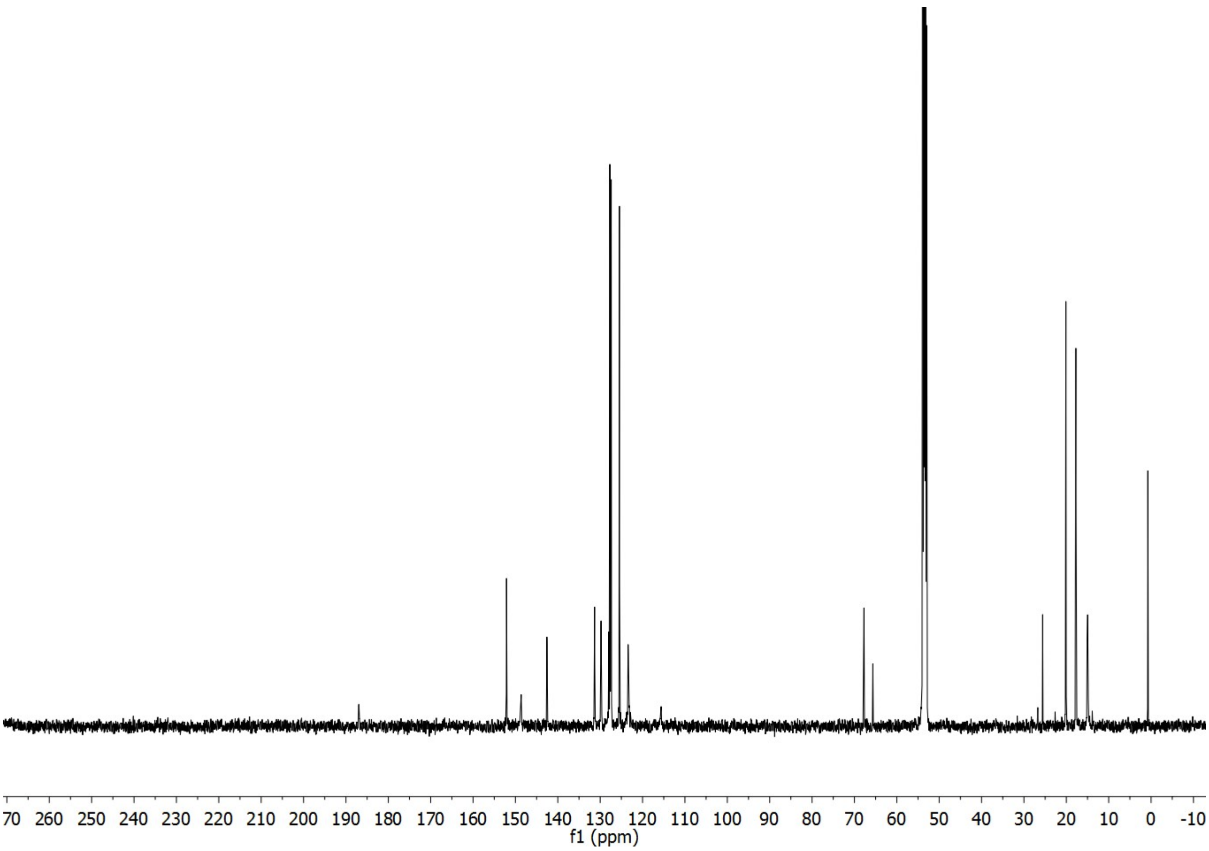
260 250 240 230 220 210 200 190 180 170 160 150 140 130 120 110 100 90 80 70 60 50 40 30 20 10 0 -10
f1 (ppm)

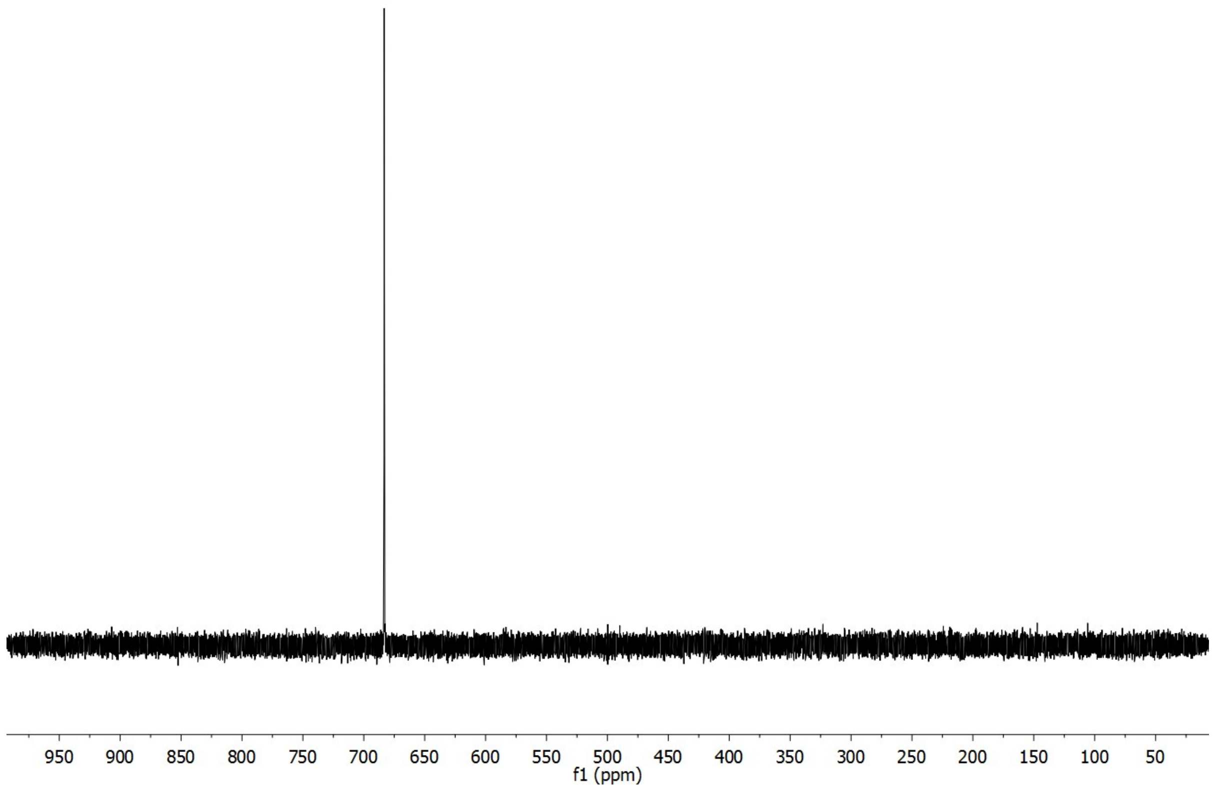

UV/Vis spectrum of compound **5** was measured in a THF solution. 2 mg of compound **5** was dissolved in 10 mL of THF. The solution was filtered over Celite and the solution was measured in a 2 mm quartz cuvette on a UV/vis/NIR lambda-19-spectrometer.

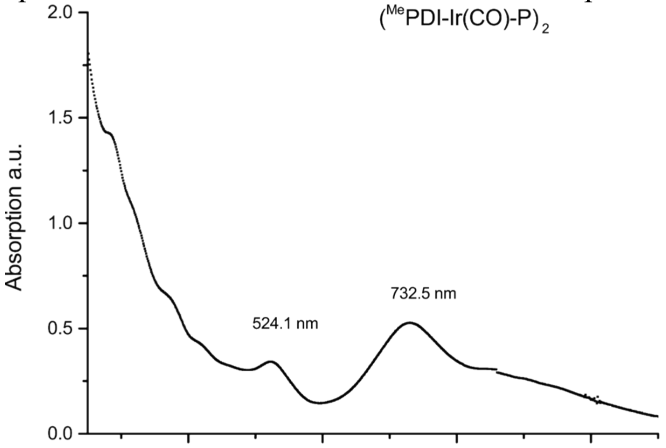

Figure S1: The UV/VIS absorption spectra of compound (**5**) was measured in a THF solution in a 2 mm quartz cuvette. The maxima were observed at 524.1 and 732.5 nm.

IR spectrum of compound 5.

The pellet was prepared with 1 mg of compound (**5**) and 50 mg of KBr. The two solids were grinded in an agate mortar and were pressed by the PIKE Technology hand press in a 7 mm die collar, in order to measure the IR spectrum on a Bruker Alpha FT-IR spectrometer (Figure S3). The CO stretching vibration $\nu_{(CO)}$ was observed at 1953 cm^{-1}. An ATIR

spectrum of the powder shows this absorption at 1967 cm^{-1}. A distinct frequency for the P=P stretching vibration could not be assigned. Several peaks are observed in the expected range between 1500 cm^{-1} and 500 cm^{-1} but most of them are related to the lattice vibration of the PDI ligand system.

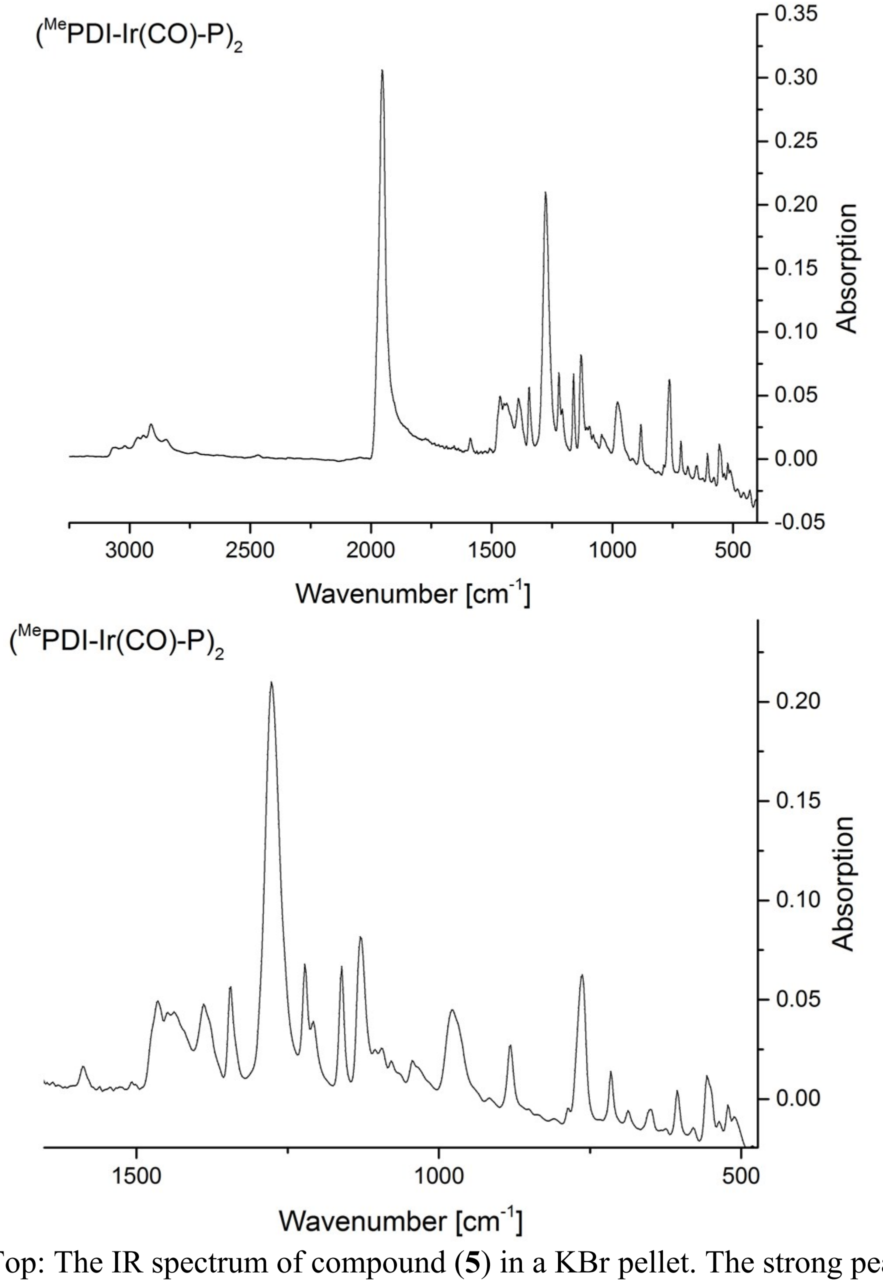

Figure S2: Top: The IR spectrum of compound (**5**) in a KBr pellet. The strong peak at 1953 cm^{-1} is assigned to the CO stretching bond $\nu_{(CO)}$. Bottom: Enlarged finger print region between 1500 cm^{-1} and 500 cm^{-1} of the IR spectrum of compound (5) (KBr pellet). Peak list [cm^{-1}]: 2910.61, 1953.02, 1464.71, 1388.65, 1344.80, 1276.27, 1221.10, 1160.70, 1129.07, 977.91, 881.78, 763.25, 715.58

Preparation of (CAAC)Au(PCO) complex 8a: A mixture of (CAAC)AuCl **6a** (1.0 g, 1.83 mmol) and [Na(PCO)(dioxane)$_{2.5}$] (0.55 g, 1.83 mmol) was cooled to -78 °C before THF (10 mL) was added. The mixture was stirred for 15 minutes and then warmed to room temperature. After 30 min, the solvent was removed under vacuum and the resulting brown solid was extracted with 15 mL of benzene. After removing the solvent, **8a** was obtained as a light yellow solid (0.73 g, yield: 70%). Colorless single crystals of **8a** were obtained by vapor diffusion of pentane into a saturated benzene solution of **8a** in the dark. IR (C_6H_6): ν PCO = 1887 cm^{-1}. M. P. = 193 °C (dec.). ^{1}H NMR (C_6D_6, 500 MHz): δ = 7.13 (t, 1 H, J = 7.3 Hz), 7.00 (d, 2 H, J = 7.3 Hz), 2.72 (sept, 2 H, J = 6.6 Hz), 1.63 (m, 4 H), 1.54 (d, 6 H, J = 6.6 Hz), 1.42 (s, 2 H), 1.08 (d, 6 H, J = 6.6 Hz), 0.85 (m, 12 H); ^{13}C{^{1}H} NMR (C_6D_6, 125 MHz): δ = 253.5 (C$_{carbene}$ d, J_{PC} = 36.2 Hz), 183.0 (C=O d, J_{PC} = 100.4 Hz), 146.1, 135.3, 130.9, 126.0, 81.0, 63.0, 43.0, 32.4, 20.1, 29.5, 27.7, 23.7, 10.3; ^{31}P{^{1}H} NMR (C_6D_6, 121 MHz) δ = -359.5. HRMS was attempted but a peak corresponding to M^{+} could not be located, probably due to the weak P metal bond.

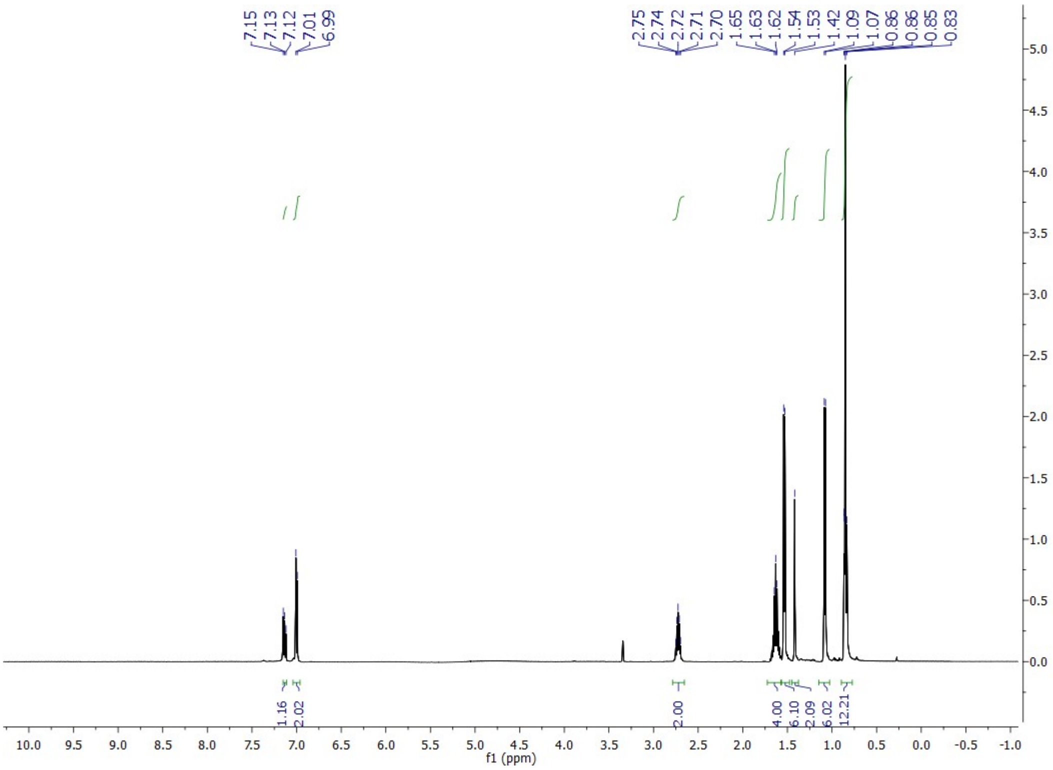

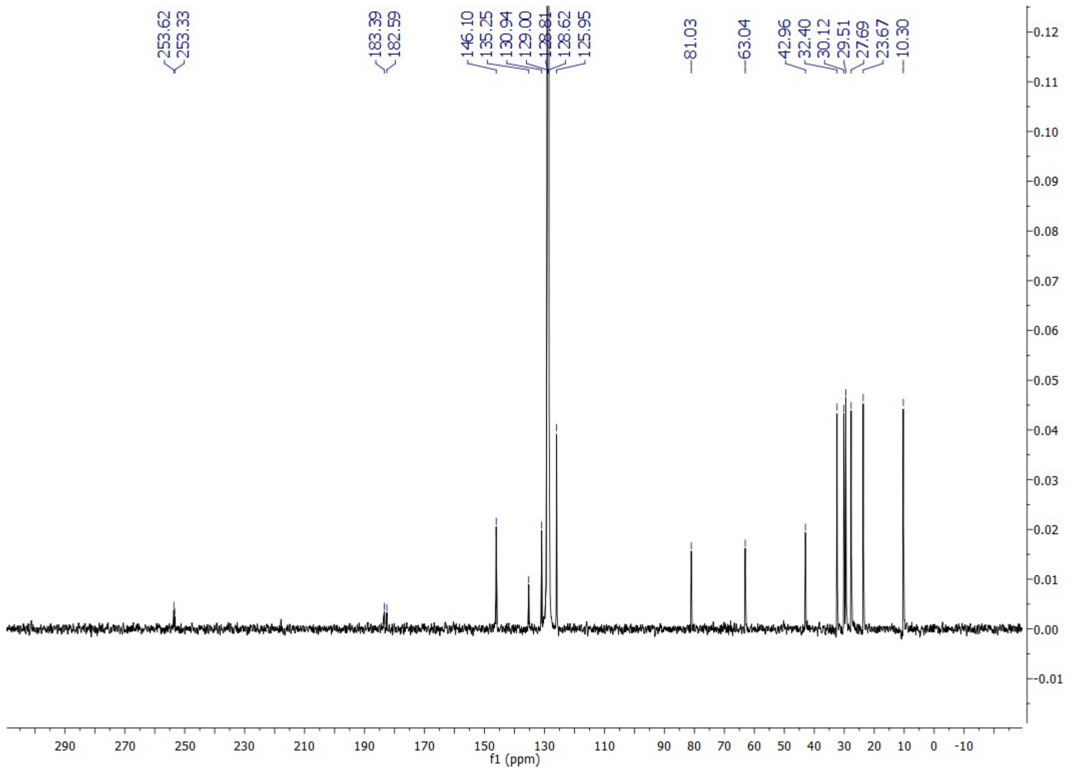

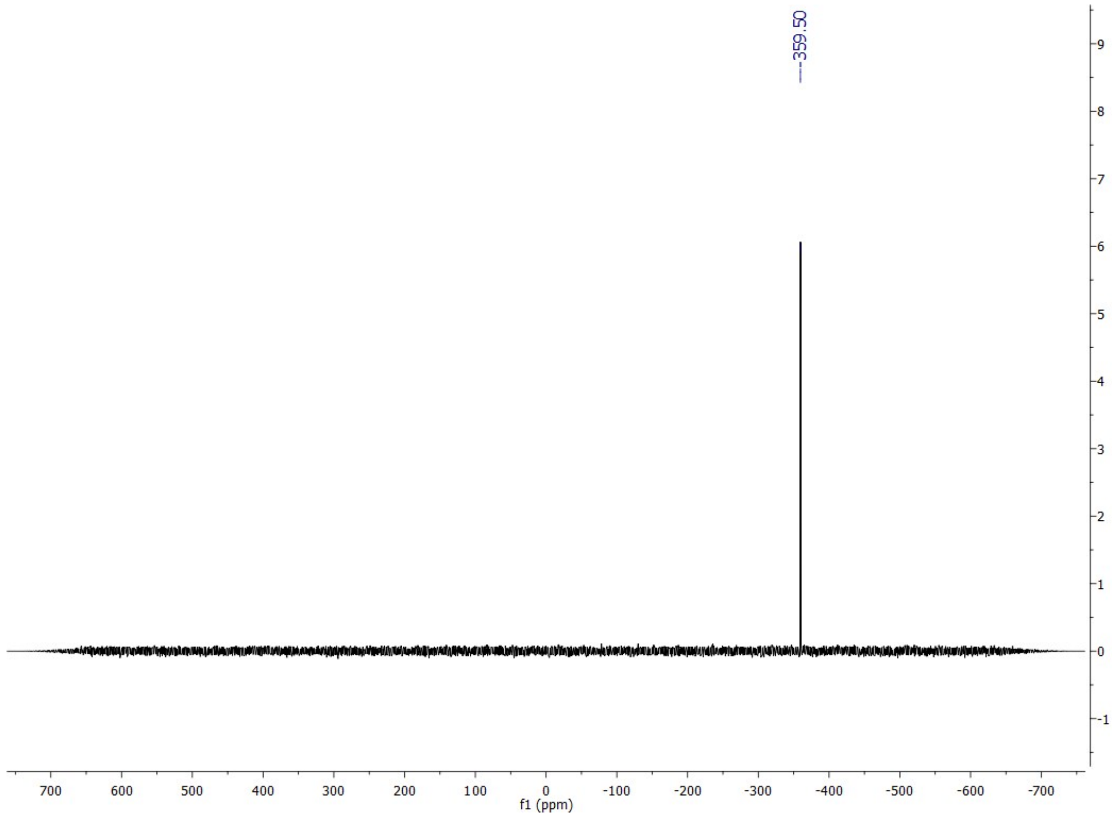

Preparation of (CAAC)Au(PCO) complex 8b: A mixture of (CAAC)AuCl **6b** (1.0 g, 1.63 mmol) and [Na(PCO) (dioxane)$_{2.5}$] (0.49 g, 1.63 mmol) was cooled to -78 °C before THF (10 mL) was added. The mixture was stirred for 15 minutes and then warmed to room temperature. After 30 min, the solvent was removed under vacuum and the resulting brown

solid was extracted with 15 mL of benzene. After removing the solvent, **8b** was obtained as a light yellow solid (0.70 g, yield: 67%). Colorless single crystals of **8b** were obtained by vapor diffusion of $(TMS)_2O$ into a saturated benzene solution of **8b** in the dark. IR (C_6H_6): ν PCO = 1889 cm^{-1}. M. P. = 221 °C (dec.). ^1H NMR (C_6D_6, 500 MHz): δ = 7.14 (br, 1 H), 7.00 (br, 2 H), 3.17 (br, 2 H), 2.79 (sept, 2 H, J = 6.8 Hz), 2.05 (m, 1 H), 1.95 (m, 1 H), 1.88 (d, 1 H, J = 12.4 Hz), 1.82 (m, 1 H), 1.69 (d, 2 H, J = 12.4 Hz), 1.58 (d, 3 H, J = 6.8 Hz), 1.52 (d, 3 H, J = 6.8 Hz), 1.26 (d, 3 H, J = 6.8 Hz), 1.09 (m, 9 H), 0.93 (m, 9 H), 0.87 (d, 3 H, J = 6.8 Hz); $^{13}C\{^1H\}$ NMR (C_6D_6, 125 MHz): δ = 253.5 ($C_{carbene}$ d, J_{PC} = 37.1 Hz), 182.5 (C=O d, J_{PC} = 101.1 Hz), 146.2, 145.8, 136.1, 130.6, 129.2, 125.8, 77.6, 65.4, 53.4, 52.0, 50.0, 36.4, 31.3, 30.1, 29.8, 28.8, 28.0, 27.3, 25.6, 23.8, 23.7, 23.6, 20.7; $^{31}P\{^1H\}$ NMR (C_6D_6, 121 MHz) δ = -364.2. HRMS was attempted but a peak corresponding to M^+ could not be located, probably due to the weak P metal bond.

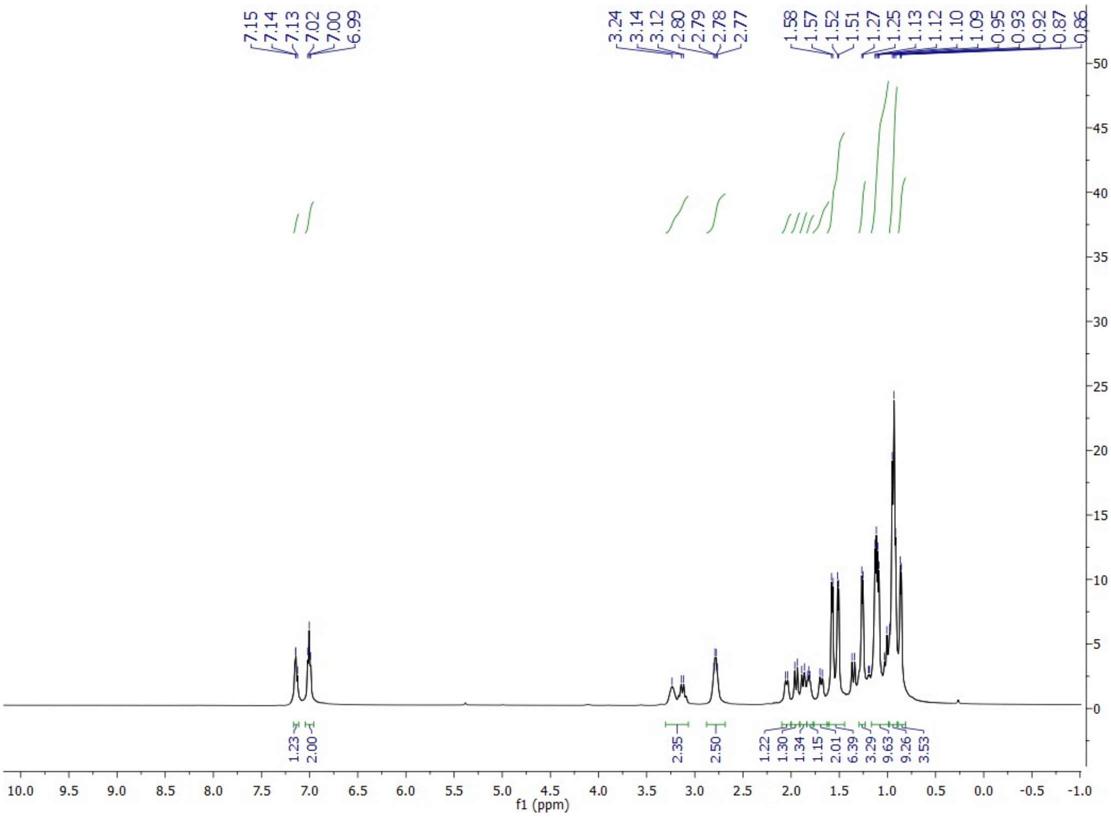

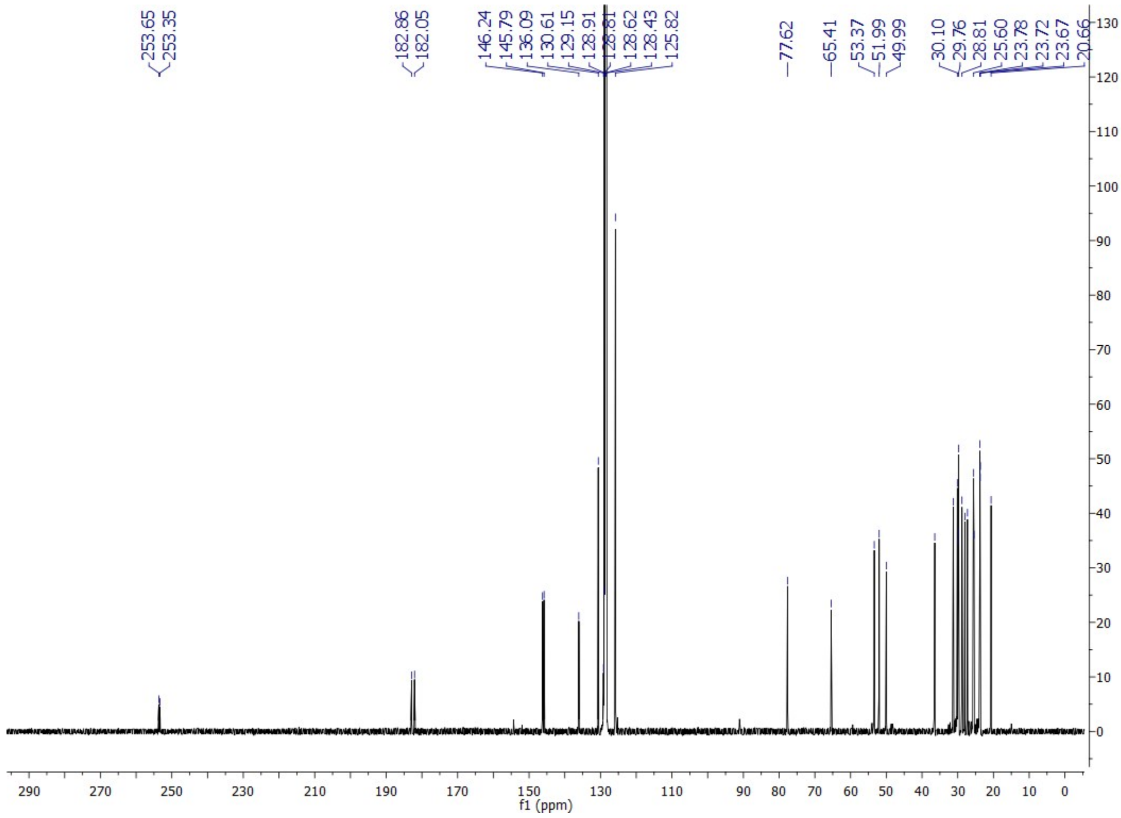

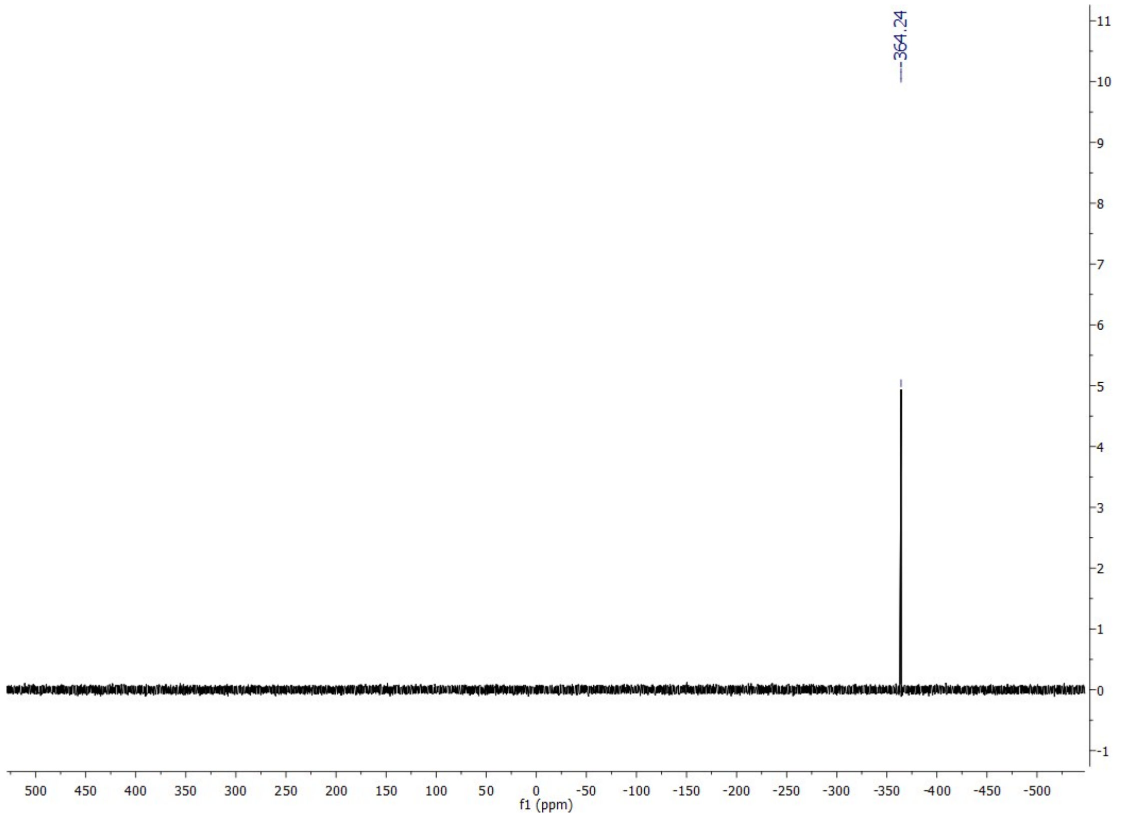

Preparation of (CAAC)Cu(PCO) complex 9: A mixture of (CAAC)CuOtBu **7** (50 mg, 0.11 mmol) and [Na(OCP) (dioxane)$_{2.5}$] (35 mg, 0.11 mmol) was stirred for 10 minutes in 3 mL of benzene at room temperature. The solvent was removed under vacuum and the resulting

brown solid was washed with 10 mL of pentane. After drying under vacuum, **9** was obtained as a light yellow solid (30 mg, yield: 62%). Colorless single crystals of **9** were obtained were obtained by vapor diffusion of $(TMS)_2O$ into a saturated toluene solution of **9** at -40 ^{o}C. IR (C_6H_6): ν PCO = 1849 cm^{-1}. M. P. = 173 °C (dec.). ^{1}H NMR (C_6D_6, 500 MHz): δ = 7.12 (t, 1 H, *J* = 7.7 Hz), 7.00 (d, 2 H, *J* = 7.7 Hz), 2.75 (sept, 2 H, *J* = 6.8 Hz), 1.68 (m, 4 H), 1.43 (d, 6 H, *J* = 6.8 Hz), 1.38 (s, 2 H), 1.08 (d, 6 H, *J* = 6.8 Hz), 0.93 (m, 6 H), 0.85 (s, 6 H); ^{13}C{^{1}H} NMR (C_6D_6, 125 MHz): δ = 251.8 ($C_{carbene}$), 175.2 (C=O d, J_{PC} = 97.8 Hz), 145.9, 135.5, 130.7, 125.6, 81.2, 63.1, 43.1, 31.9, 29.9, 29.4, 27.9, 23.1, 10.4; ^{31}P{^{1}H} NMR (C_6D_6, 121 MHz) δ = -387.4. HRMS was attempted but a peak corresponding to M^{+} could not be located, probably due to the weak P metal bond.

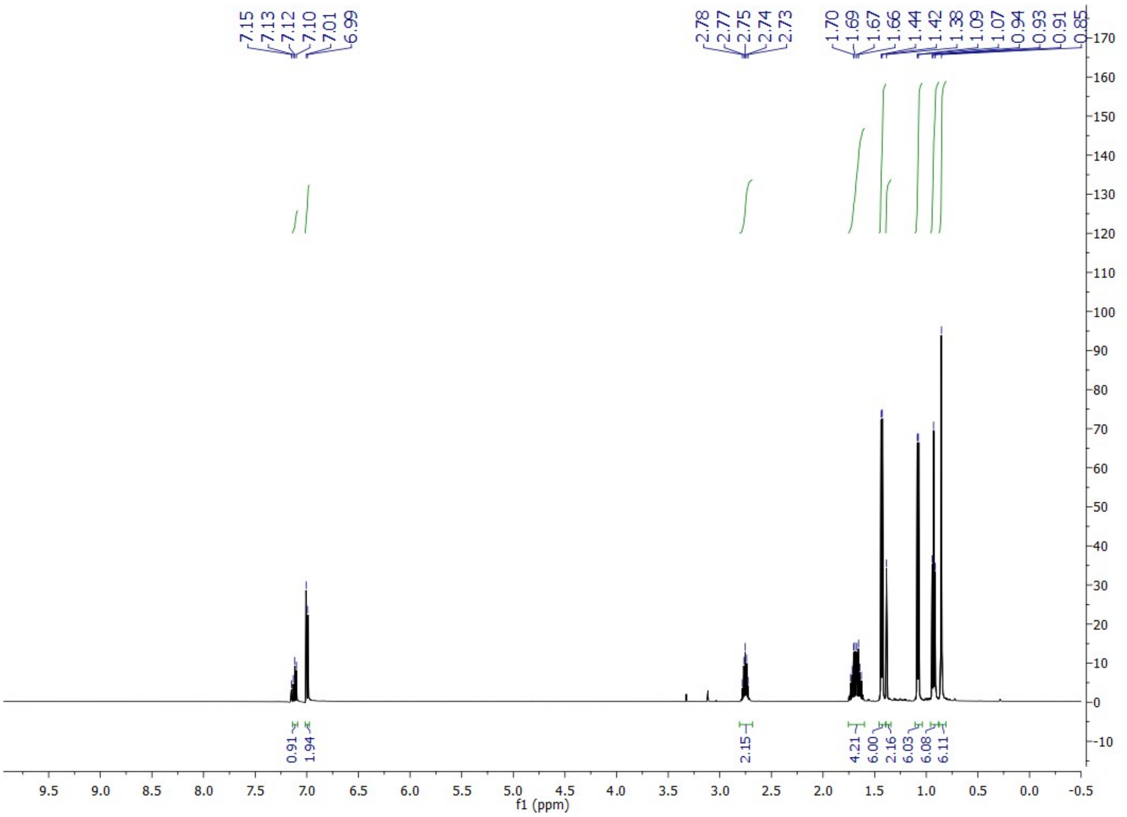

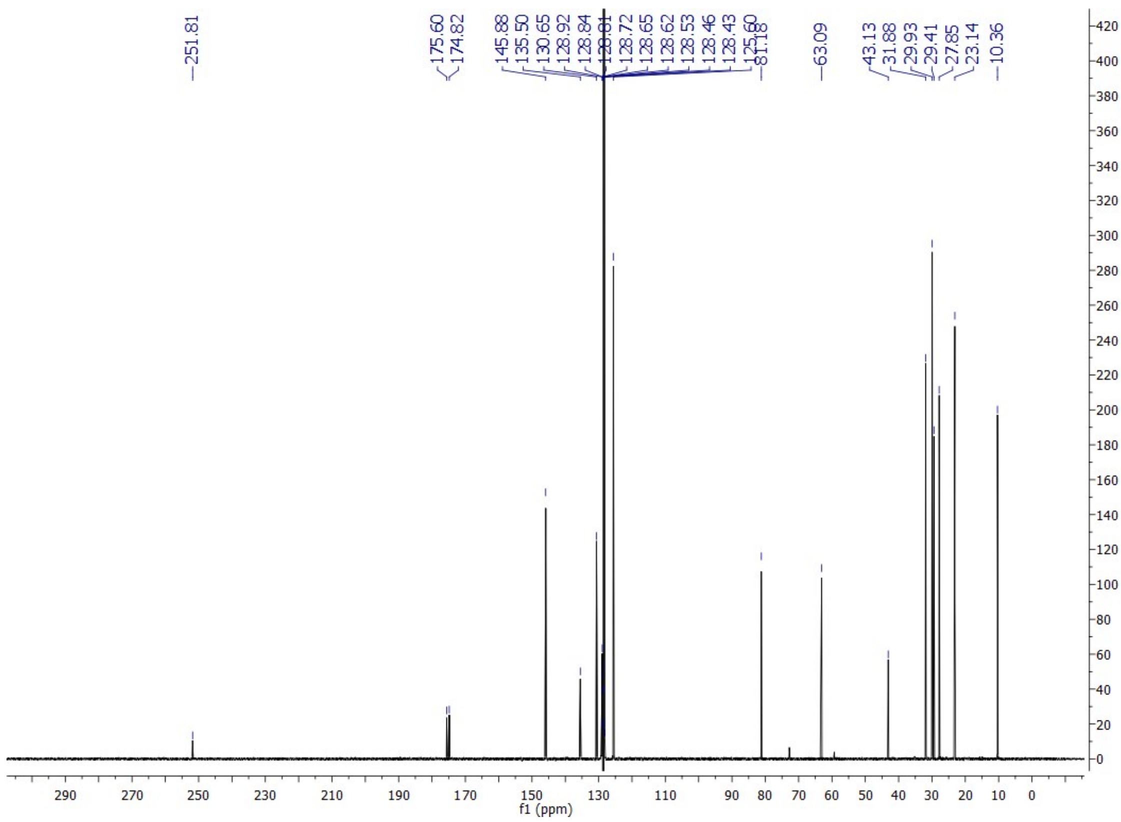

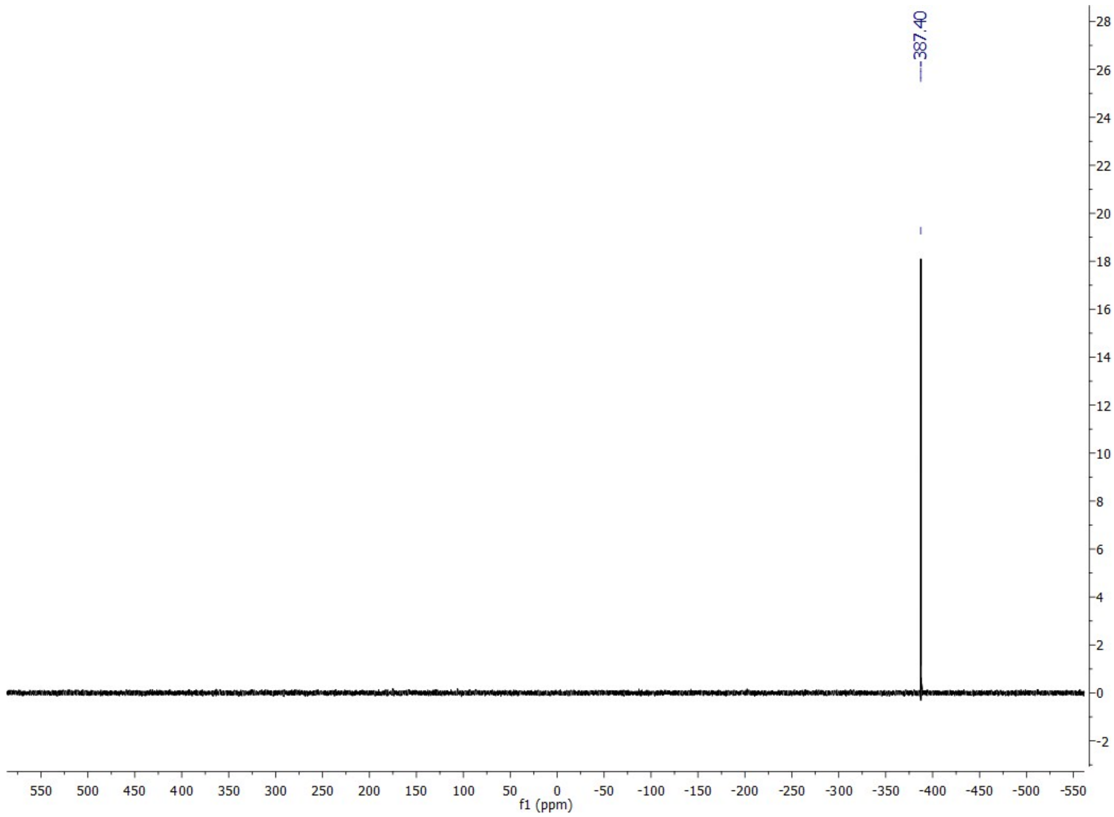

Preparation of ($L_bAu)_3P$ 10: Complex **8b** (50 mg, 0.078 mmol) was left standing in 2 mL of THF for 1 week under visible light. White crystals of **10** were generated and washed with 5 mL of pentane (10 mg, yield: 22%). M. P. = 293 °C (dec.). ^1H NMR (CD_2Cl_2, 500 MHz): δ =

7.39 (t, 3 H, J = 8.4 Hz), 7.18 (m, 6 H), 2.72 (m, 12 H), 2.28 (m, 3 H), 1.88 (m, 9 H), 1.78 (m, 3 H), 1.67 (m, 3 H), 1.34 (m, 9 H), 1.24 (m, 36 H), 1.13 (d, 9 H, J = 6.6 Hz), 0.98 (m, 18 H), 0.90 (d, 9 H, J = 6.6 Hz), 0.82 (d, 9 H, J = 6.6 Hz); $^{13}C\{^1H\}$ NMR (CD_2Cl_2, 125 MHz): δ = 260.3 ($C_{carbene}$ d, J_{PC} = 84 Hz), 146.3, 145.7, 135.3 130.2, 125.5, 125.2, 79.0, 78.9, 66.5, 66.4, 52.9, 50.5, 36.5, 30.5, 30.4, 30.0, 29.9, 29.5, 29.0, 28.9, 28.5, 25.2, 25.1, 24.5, 23.3 ,23.2, 21.3; $^{31}P\{^1H\}$ NMR (CD_2Cl_2, 121 MHz) δ = -200.2. HRMS: m/z calculated for $[C_{81}H_{130}N_3Au_3P]^+$ $(M+H)^+$ 1766.8999; found 1766.8980.

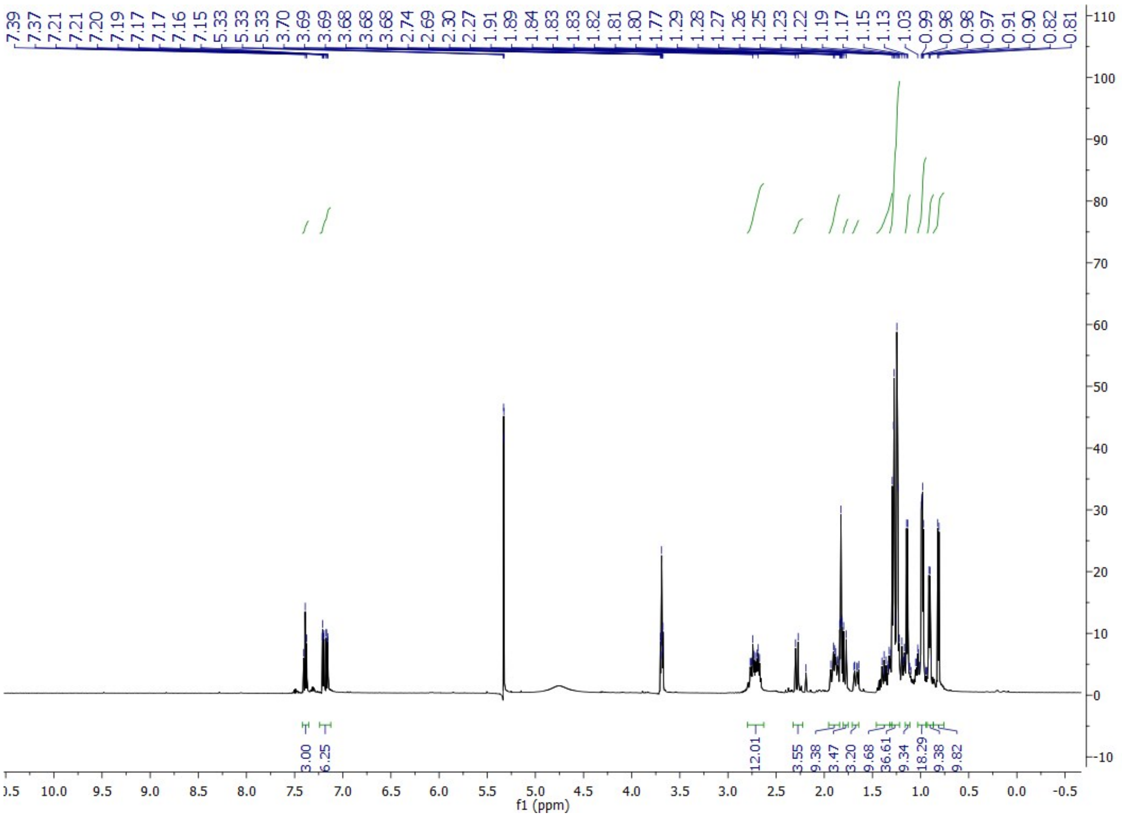

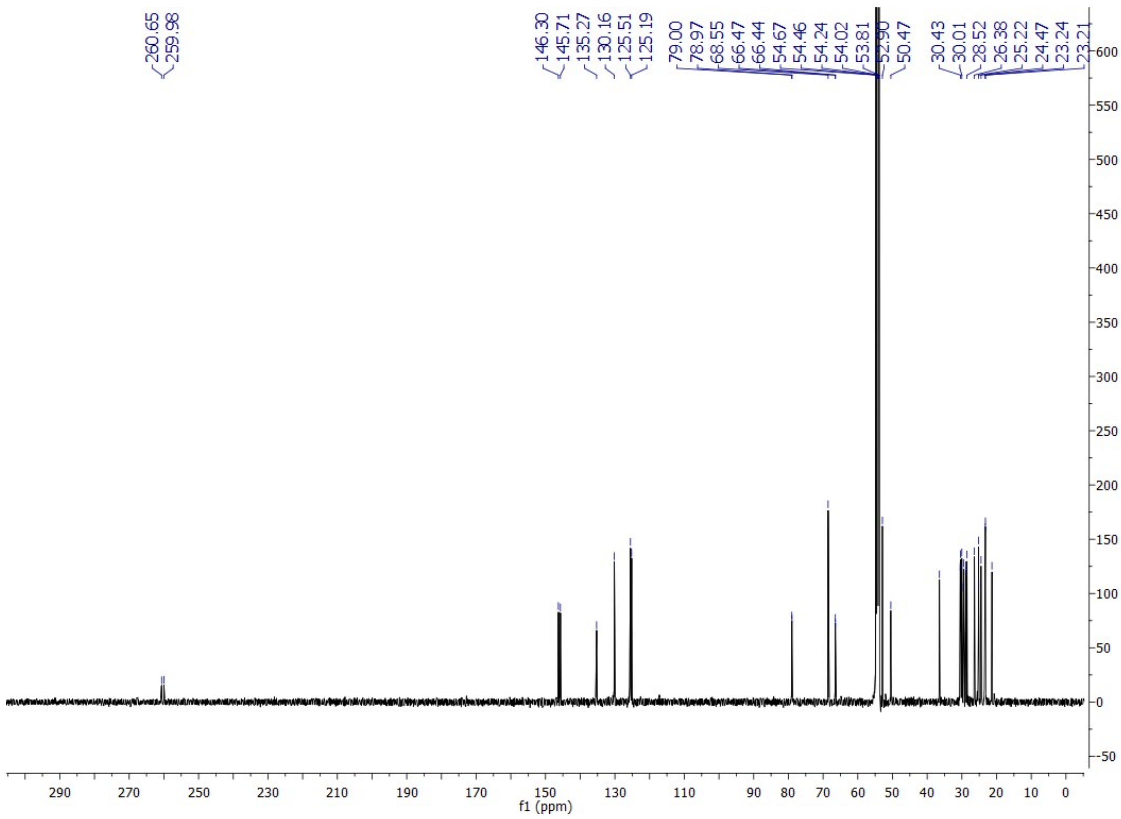

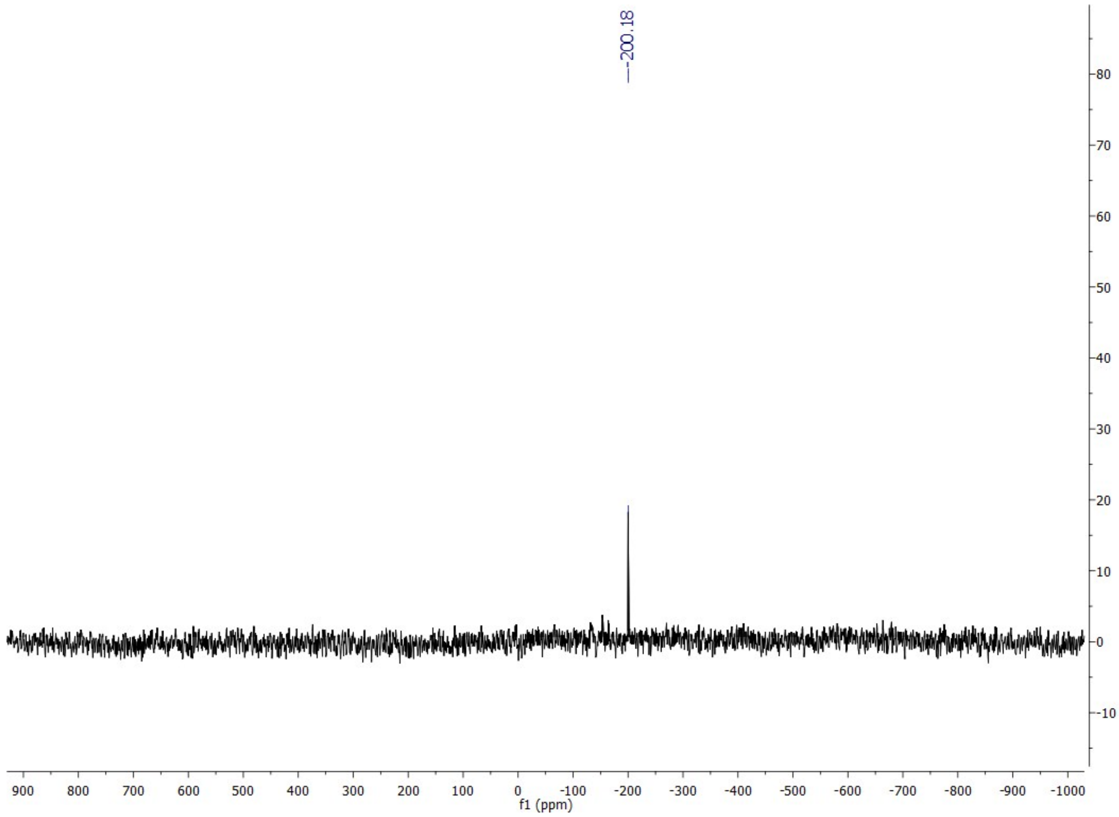

Preparation of bis(CAAC)Cu$^+$PCO$^-$ complex 11: A mixture of **9** (20 mg, 0.046 mmol) and carbene L_a (15 mg, 0.049 mmol) was stirred for 2 min in benzene (0.5 mL). The suspension was filtered and the colorless powder was washed with benzene (1 mL), yielding **11** (30 mg,

86% yield). Single crystals were obtained by slow evaporation of a saturated benzene solution of **11**. IR (solid, KBr): PCO ν = 1791 cm^{-1}. M. P. = 158 °C (dec.). ^{1}H NMR (C_6D_6, 500 MHz): δ = 7.08 (t, 2 H, *J* = 7.8 Hz), 6.97 (d, 4 H, *J* = 7.8 Hz), 2.70 (sept, 4 H, *J* = 6.8 Hz), 1.54 (m, 8 H), 1.42 (d, 12 H, *J* = 6.8 Hz), 1.32 (s, 4 H), 1.05 (d, 12 H, *J* = 6.8 Hz), 0.84 (t, 12 H, *J* = 7.2 Hz), 0.81 (s, 12 H); ^{13}C{^{1}H} NMR (C_6D_6, 125 MHz): δ = 253.3 ($C_{carbene}$), 145.8, 135.3, 130.7, 125.5, 80.8, 63.0, 43.6, 31.6, 30.0, 29.4, 28.0, 23.1, 10.2; ^{31}P{^{1}H} NMR (C_6D_6, 121 MHz) δ = -399.5. HRMS: m/z calculated for $[C_{44}H_{70}CuN_2]^+$ 689.4835; found 689.4847.

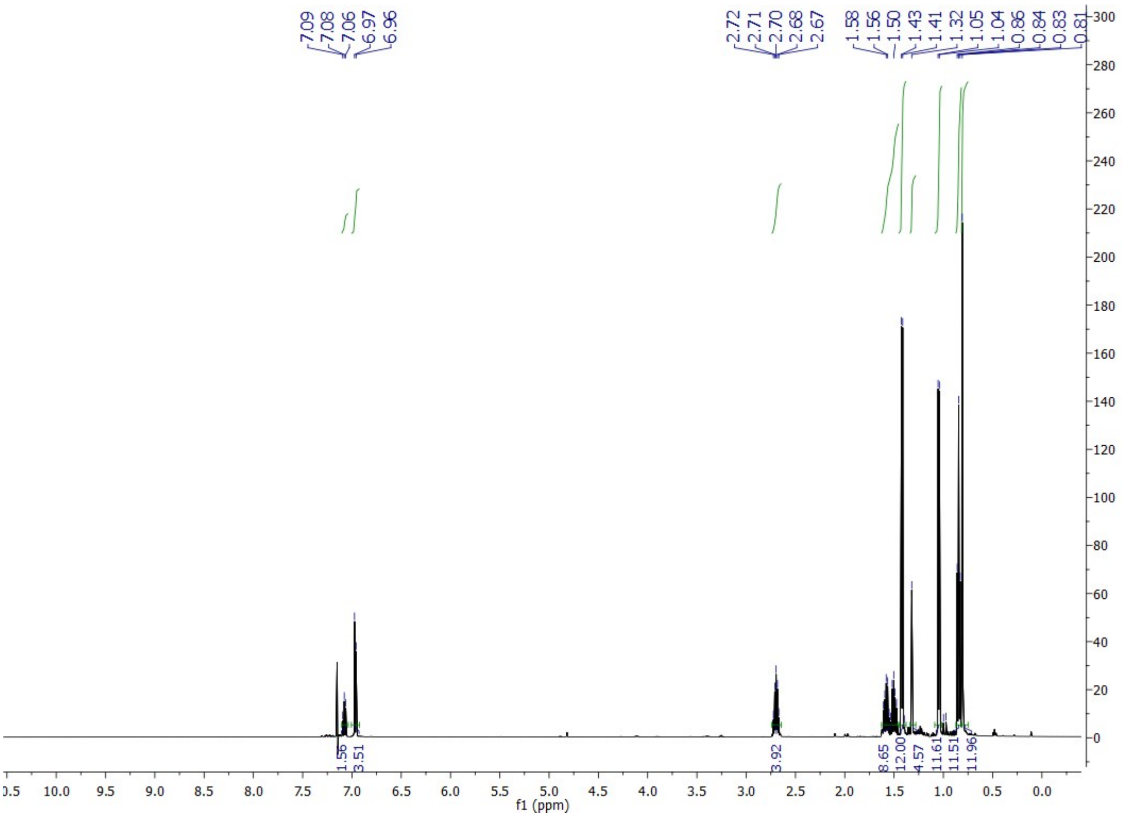

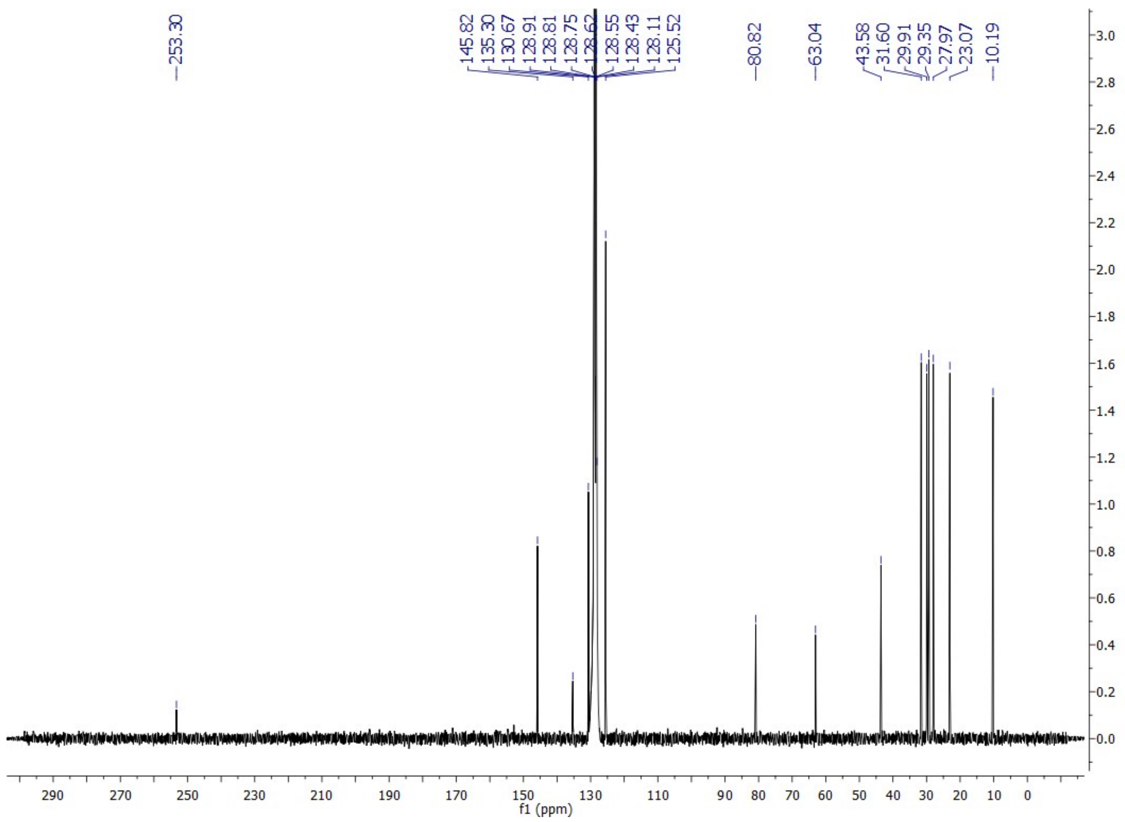

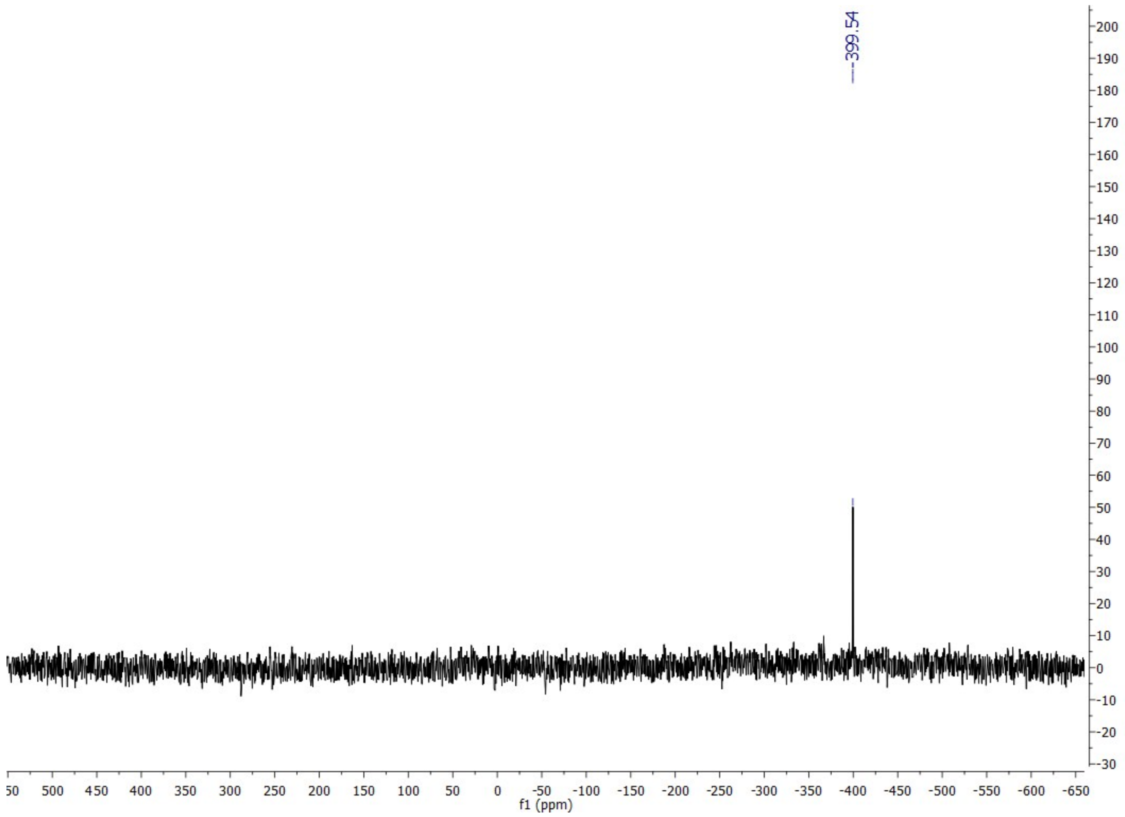

Preparation of four-membered heterocycle 12: A mixture of **8b** (50 mg, 0.078 mmol) and $B(C_6F_5)_3$ (40 mg, 0.078 mmol) was stirred in 2 mL of benzene for 5 minutes. The resulting yellow suspension was filtered and the yellow residue was washed with benzene (0.5 mL),

then dried under vacuum, yielding 61 mg (64%) of a bright-yellow powder. Yellow single crystals of **12** were obtained in the filtrate in less than 1 min. M. P. = 230 °C (dec.).

Preparation of four-membered heterocycle 13: A mixture of **9** (50 mg, 0.12 mmol) and $B(C_6F_5)_3$ (59 mg, 0.12 mmol) was stirred in 1 mL of toluene for 5 minutes. The solvent was removed under reduced pressure and the residue was washed with pentane (2 mL), yielding 68 mg (62 %) of a light-yellow powder. Colorless single crystals were obtained by vapor diffusion of $(TMS)_2O$ into a saturated toluene solution of **13**. M. P. = 160 °C (dec.). ^{1}H NMR (C_6D_6, 500 MHz): δ = 7.08 (t, 2 H, J = 8.1 Hz), 6.93 (d, 4 H, J = 8.1 Hz), 2.59 (sept, 4 H, J = 6.6 Hz), 1.40 (m, 8 H), 1.31 (m, 4 H), 1.12 (d, 12 H, J = 6.6 Hz), 1.08 (d, 12 H, J = 6.6 Hz), 1.01 (m, 12 H), 0.85 (m, 12 H); ^{13}C{^{1}H} NMR (C_6D_6, 125 MHz): δ = 248.8 ($C_{carbene}$ br), 149.5 (br d, J_{FC} = 238 Hz), 149.0 (br d, J_{FC} = 247 Hz), 145.5, 138.1 (br d, J_{FC} = 247 Hz), 135.1, 131.2, 125.8, 82.5, 62.8, 43.0, 31.5, 29.7, 29.2, 27.7, 22.7, 9.9; ^{31}P{^{1}H} NMR (C_6D_6, 121 MHz) δ = 260.6 (br), 136.2 (br). HRMS was attempted but a peak corresponding to M^+ could not be located, probably due to the weak P metal bond.

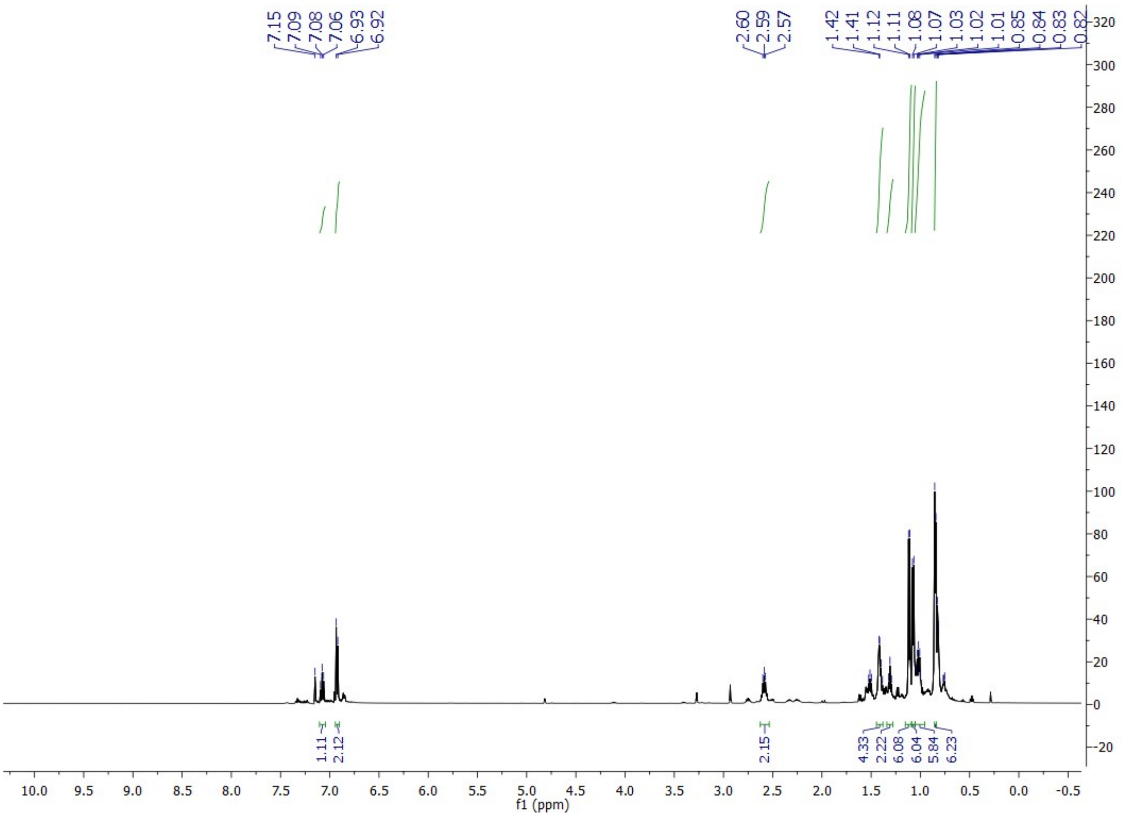

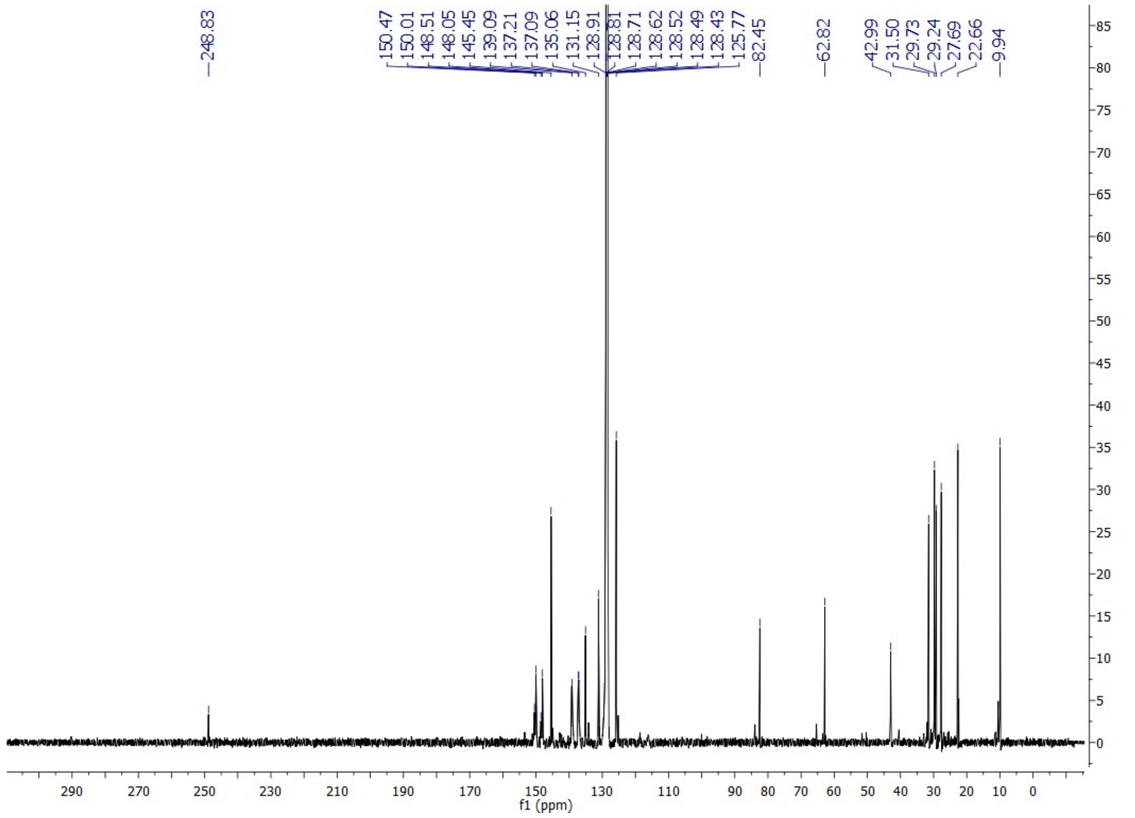
248.83
150.47
150.01
148.51
148.05
145.45
139.09
137.21
137.09
135.06
131.15
128.91
128.81
128.71
128.62
128.52
128.49
128.43
125.77
82.45
62.82
42.99
31.50
29.73
29.24
27.69
22.66
9.94
f1 (ppm)

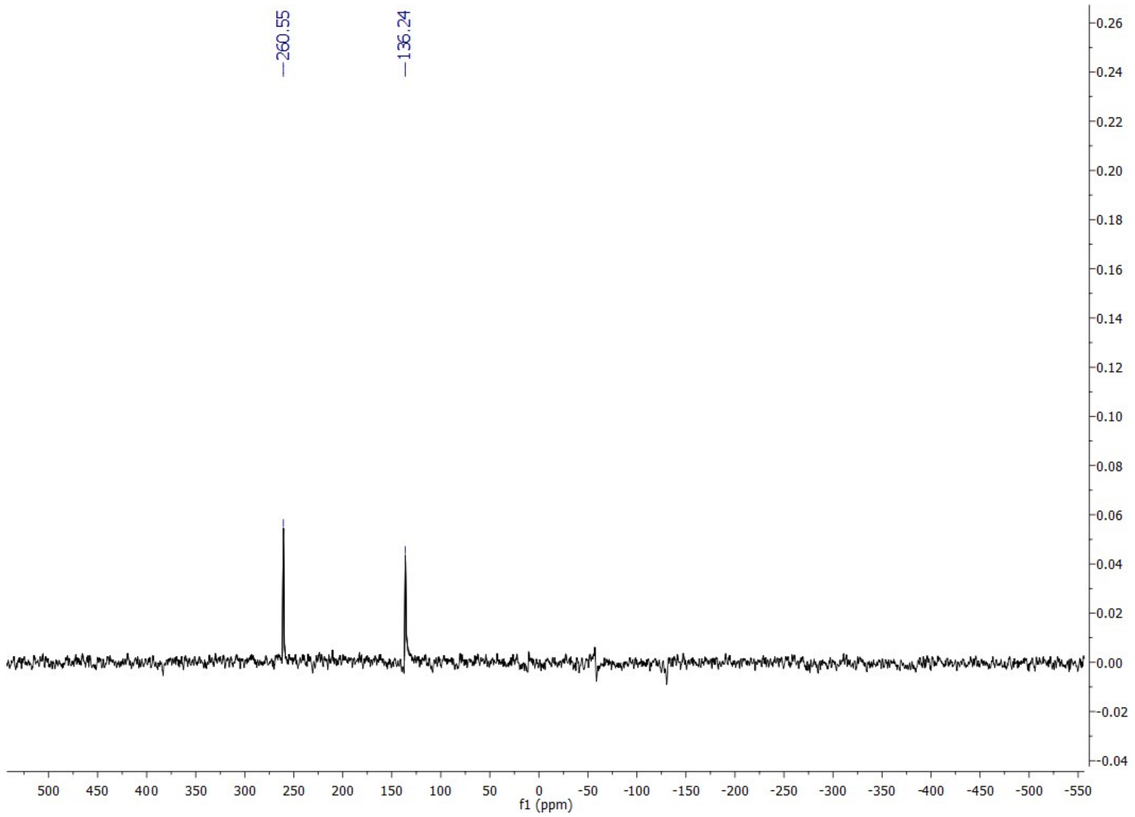
260.55
136.24
f1 (ppm)

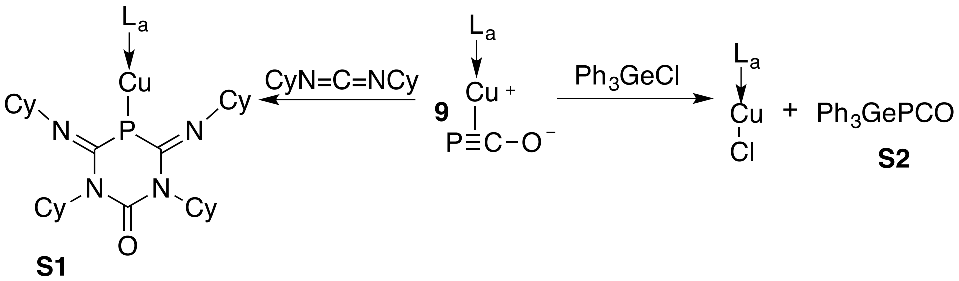

Preparation of S1: A mixture of **9** (50 mg, 0.12 mmol) and CyNCNCy (50 mg, 0.24 mmol) was stirred in 1 mL of benzene for 30 minutes at 50 °C. The solvent was removed under reduced pressure and the residue was extracted with pentane (5 mL), yielding 73 mg (72%) of yellow **S1**. Colorless single crystals of **S1** were obtained by slow evaporation of a saturated pentane solution. M. P. = 170 °C (dec.). ^{1}H NMR (C_6D_6, 500 MHz): δ = 7.04 (t, 1 H, J = 7.7 Hz), 6.90 (d, 2 H, J = 7.7 Hz), 5.13 (m, 2 H), 3.77 (m, 2 H), 3.01 (m, 4 H), 2.65 (m, 4 H), 2.00-1.27 (overlapping m, 46 H), 1.06 (d, 6 H, J = 6.8 Hz), 0.94 (t, 6 H, J = 7.2 Hz), 0.75 (s, 6 H); $^{13}C\{^{1}H\}$ NMR (C_6D_6, 125 MHz): δ = 252.0 ($C_{carbene}$ d, J_{PC} = 28.8 Hz), 162.2 (C=N d, J_{PC} = 46.2 Hz), 155.2 (C=O), 145.5, 134.9, 130.7, 130.5, 125.4, 125.3, 81.7, 63.5, 60.5, 60.4, 60.3, 60.2, 58.8, 58.6, 42.6, 35.1 (br), 31.8, 31.2, 31.1, 30.0, 29.9, 29.3, 29.2, 28.6 (br), 27.4 (br), 25.8 (br), 22.7, 22.6, 10.4, 10.3. $^{31}P\{^{1}H\}$ NMR (C_6D_6, 121 MHz) δ = -88.8. HRMS: m/z calculated for $[C_{49}H_{80}N_5OPCu]^+$ $(M+H)^+$ 848.5391; found 848.5408.

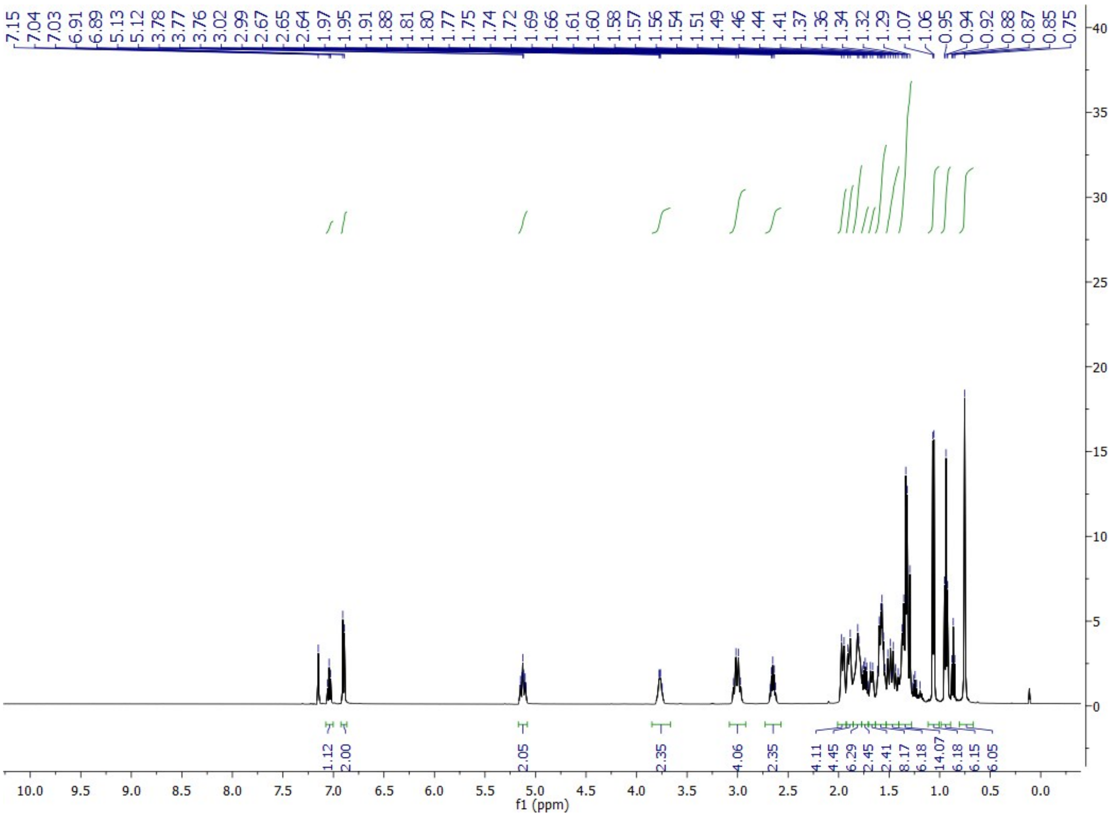

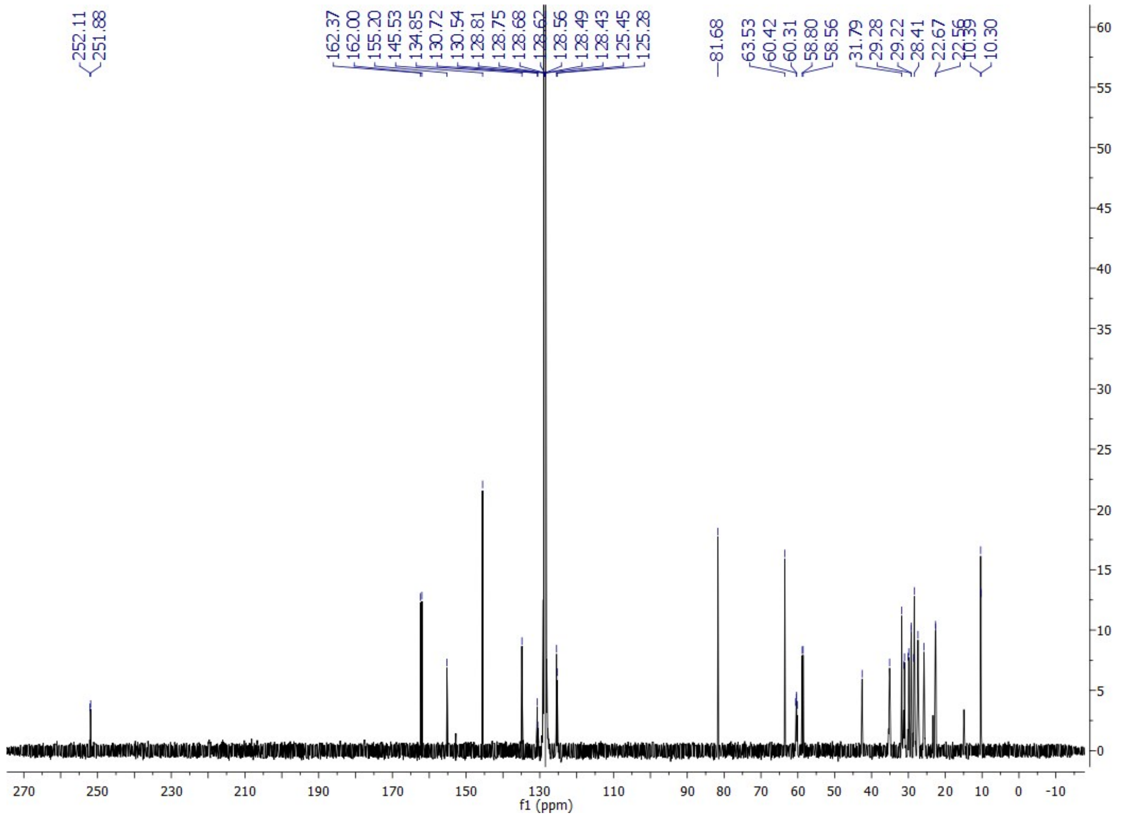

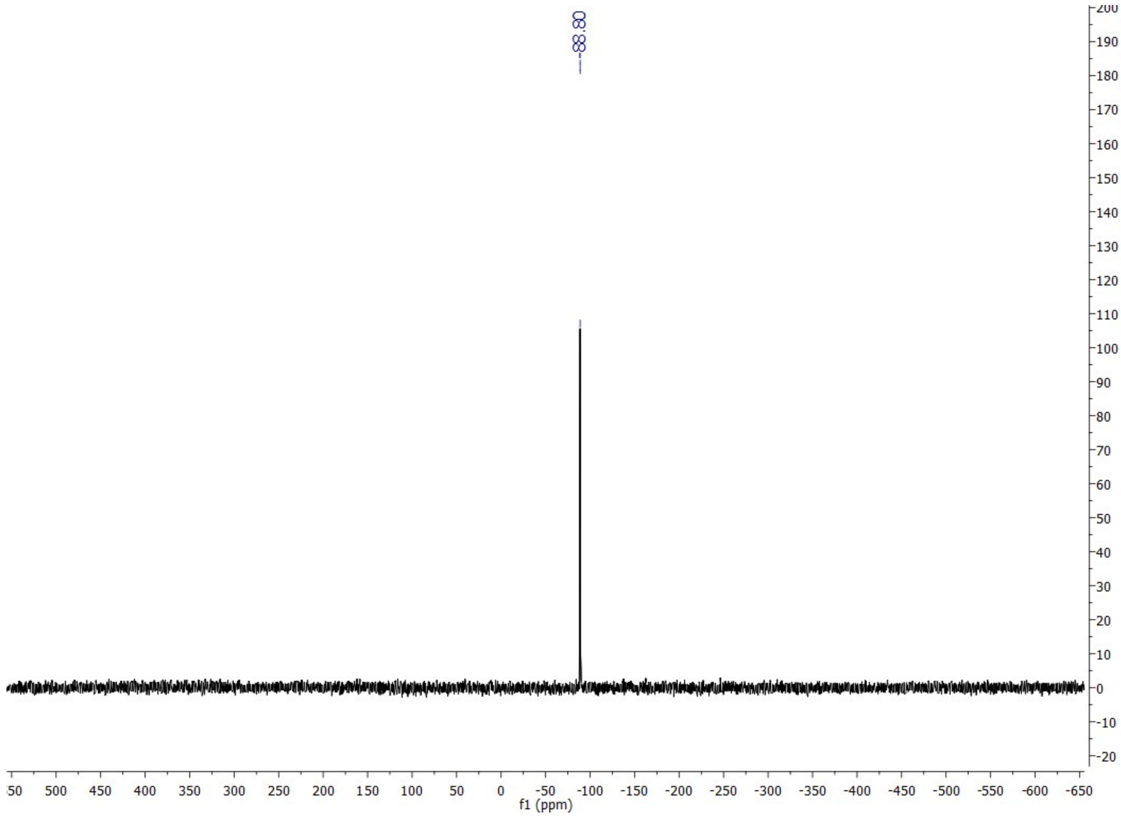

Preparation of S2: A mixture of **9** (20 mg, 0.046 mmol) and Ph_3GeCl (16 mg, 0.046 mmol) was stirred for 2 min in toluene (0.5 mL). The NMR spectra are identical as previous reports assigned to **S2** and CAACCuCl.

Crystallographic Data

	**8a**	**8b**	**9**	**10**
Formula	$C_{46}H_{70}N_2O_2Au_2P_2$	$C_{28}H_{43}AuNOP$	$C_{23}H_{35}CuNOP$	$C_{40.5}H_{64.5}Au_{1.5}N_{1.5}P_{0.5}$
wt	1138.91	637.57	436.03	883.37
Cryst. syst.	Monoclinic	Orthorhombic	Monoclinic	Hexagonal
Space group	$P2_1/n$	$P2_12_12_1$	$P2_1/n$	R32
a(Å)	9.3038(5)	10.7215(7)	9.1985(7)	23.2392(6)
b(Å)	14.9607(8)	13.5360(7)	15.0111(11)	23.2392(6)
c(Å)	16.6489(9)	19.0499(12)	16.6696(13)	27.9791(10)
□(deg)	90.00	90.00	90.00	90.00
□(deg)	91.195(2)	90.00	90.736(3)	90.00
□(deg)	90.00	90.00	90.00	120.00
V($Å^3$)	2316.9(2)	2764.6(3)	2301.5(3)	13086.0(7)
Z	2	4	4	12
d(calc) gcm^{-3}	1.633	1.532	1.258	1.345
R(int)	0.0423	0.0534	0.0473	0.0708
□, mm^{-1}	6.431	5.398	2.071	5.089
Total data	4255	4873	4181	5380
$>2\sigma(F_O^2)$	3640	4652	4057	4659
Variables	252	281	252	274
R (>2□)	0.0203	0.0358	0.0255	0.0384
R_w	0.0426	0.0666	0.0689	0.0899
GOF	1.023	1.321	1.050	1.027

	**S1**	**11**	**12**	**13•(C_7H_8)$_2$**
Formula	$C_{98}H_{158}Cu_2N_{10}O_2P_2$	$C_{46}H_{70}CuN_2O_2P$	$C_{46}H_{43}AuBF_{15}NOP$	$C_{384}H_{344}B_8Cu_8F_{120}N_8O_8P_8$
wt	1697.36	777.55	1149.56	8321.23
Cryst. syst.	Monoclinic	Monoclinic	Tetragonal	Monoclinic
Space group	$P2_1/n$	$P2_1/n$	P4(3)2(1)2	C2/c
a(Å)	12.6627(14)	9.4619(4)	14.7478(2)	26.7657(13)
b(Å)	16.2794(15)	13.4134(6)	14.7478(2)	16.5433(13)
c(Å)	23.264(3)	16.9001(8)	40.2121(10)	21.4790(13)
□(deg)	90.00	90.00	90.00	90.00
□(deg)	98.409(3)	101.411(2)	90.00	107.104(3)

□(deg)	90.00	90.00	90.00	90.00
V(Å^3)	4744.1(9)	2102.50(16)	8746.0(3)	9090.1(10)
Z	2	2	8	1
d(calc) gcm^{-3}	1.188	1.228	1.746	1.520
R(int)	0.0787	0.1005	0.0815	0.0629
□, mm^{-1}	0.534	0.596	3.503	0.616
Total data	8282	3706	7708	8353
>2σ(F_O^2)	4839	3001	6379	6701
Variables	522	234	605	623
R (>2□)	0.0632	0.0893	0.0437	0.0347
R_w	0.1379	0.2121	0.0952	0.1059
GOF	1.029	1.249	1.131	1.098

	**3**	**5**
Formula	$C_{42}H_{59}CoN_3O_3P$	$C_{30}H_{35}IrN_3O_2P$
wt	743.82	692.8
Cryst. syst.	Triclinic	Monoclinic
Space group	P-1	P2$_1$/n
a(Å)	10.1044(5)	13.7808(13)
b(Å)	14.1614(7)	12.3485(16)
c(Å)	15.8020(7)	16.5685(19)
□(deg)	111.476(4)	90.00
□(deg)	93.948(4)	96.530(4)
□(deg)	104.766(4)	90.00
V(Å^3)	2001.36(16)	2801.2(6)
Z	2	4
d(calc) gcm^{-3}	1.234	1.643
R(int)	0.0727	0.0433
□, mm^{-1}	0.509	4.855
Total data	30723	78327
>2σ(F_O^2)	8175	13573
Variables	507	340
R (>2□)	0.0699	0.0321
R_w	0.1304	0.0511
GOF	1.158	1.937

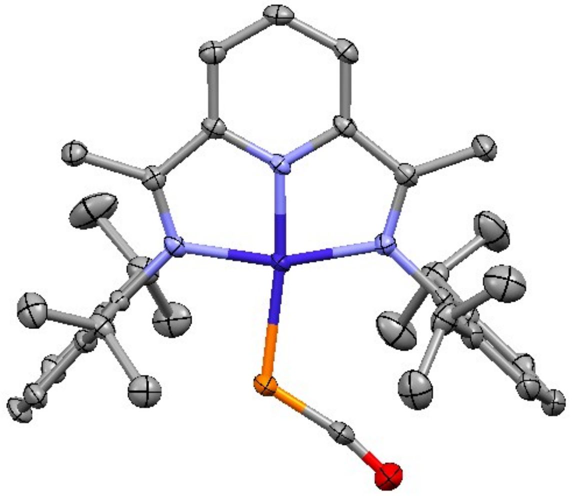

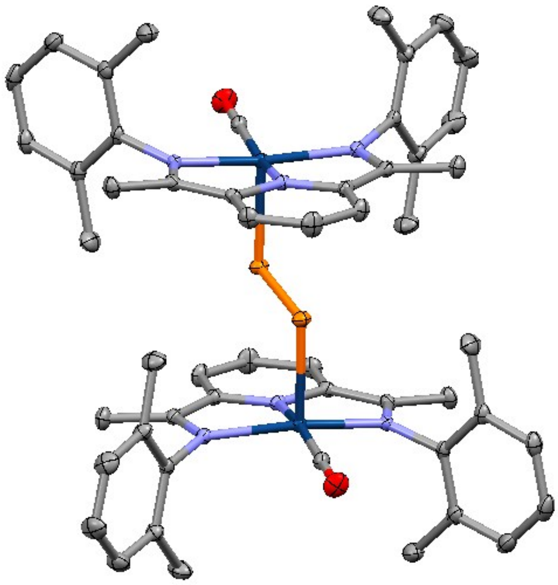

Molecular view of **3** and **5** (50% thermal ellipsoids are shown) with hydrogen atoms omitted for clarity.

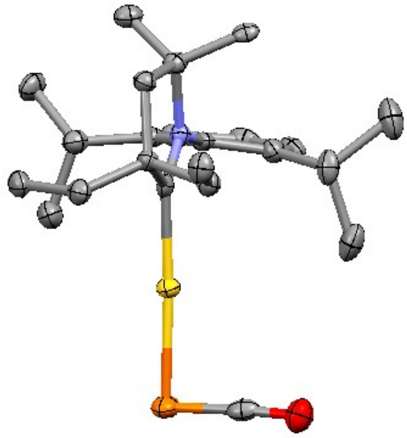

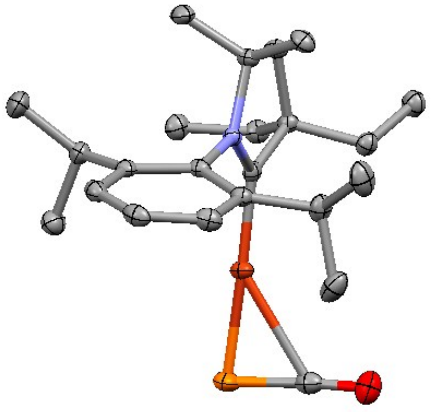

Molecular view of **8a** and **9** (50% thermal ellipsoids are shown) with hydrogen atoms omitted for clarity.

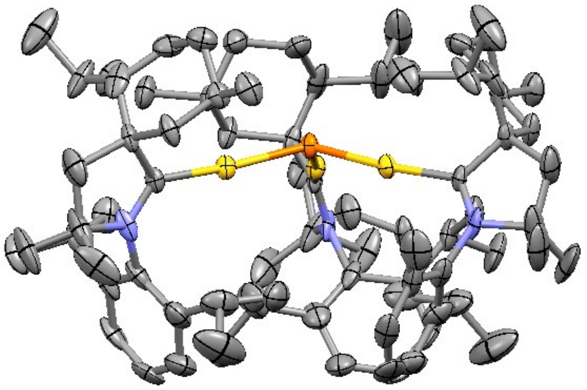

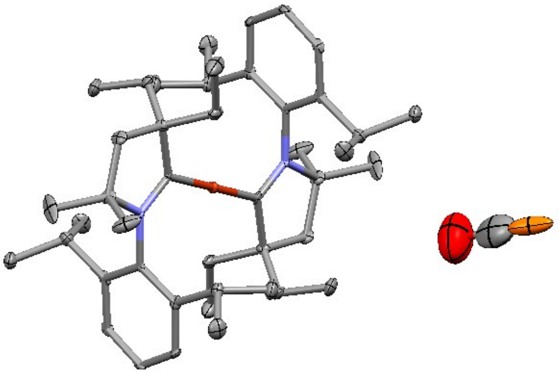

Molecular view of **10** and **11** (50% thermal ellipsoids are shown) with hydrogen atoms omitted for clarity.

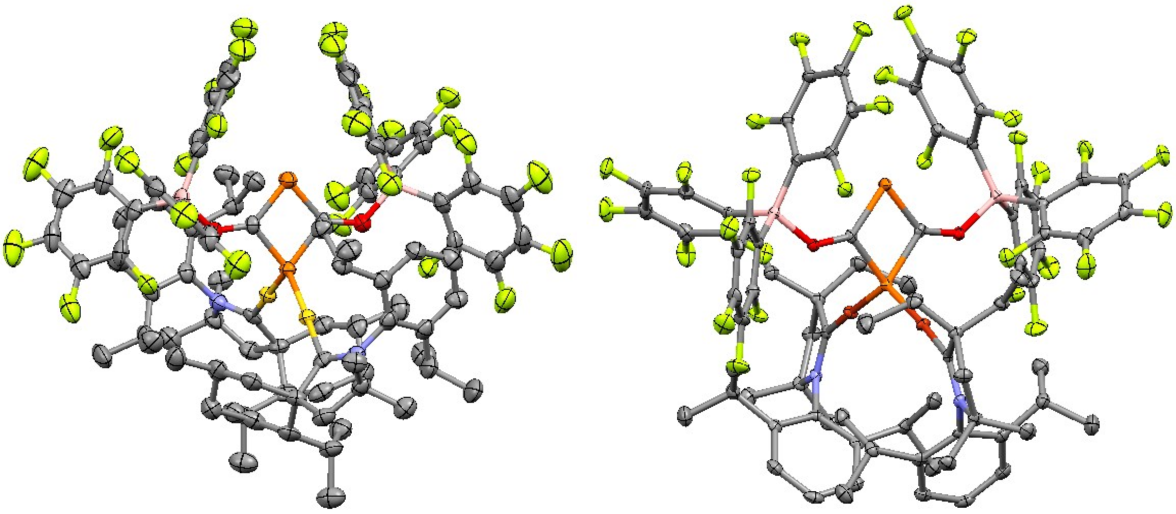

Molecular view of **12** and **13** (50% thermal ellipsoids are shown) with hydrogen atoms omitted for clarity.

Computational Details

Calculations were carried out with the Gaussian 09 package.[1] Geometry optimizations were performed with the M06 functional.[2] A mixed basis set employing 6-31G(d)[3] for C, H, O, N and P atoms and LANL2DZ[4] for Au and Cu was used. Polarization functions were added for Au (ξ_f = 1.05) and Cu (ξ_f = 3.525).[5] Frequency calculations at the same level of theory were performed to identify the number of imaginary frequencies (zero for local minimum) and provide frontier molecular orbitals (HOMO). A larger mixed basis set employing 6-311++G(2d,p)[6] for C, H, O, N and P atoms and SDD[7] for Au and Cu was used for Natural bond orbital (NBO) calculations using NBO 3.0 program.

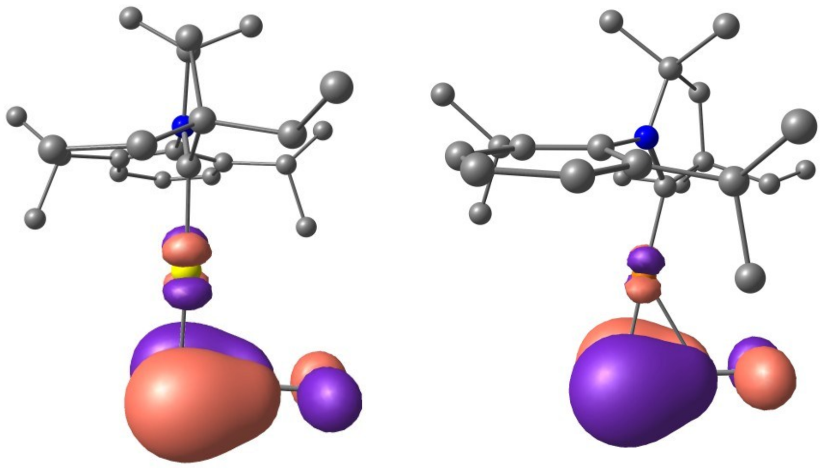

HOMOs of **8a** (left) and **9** (right) (isovalue = 0.05 a.u.)

The geometry of the complex **5** was optimized starting from the crystal structure data using Gaussian 09[1] employing lanl2dz (Ir)[2] and b3lyp/6-31G* (C, H, N, O, P)[3] as functionals. The calculated IR spectrum is shown in Figure S3. The wave number for the P=P stretching vibration is 498.21 cm^{-1} but the dipole strength is 0 10^{-40} esu^2 cm^2. The P=P frequency likely is IR inactive and explains why this band was not found experimentally. The calculated CO stretching vibration [$\nu_{(CO)}^{calc}$ = 1937 cm^{-1}] is in good agreement with the experimental data.

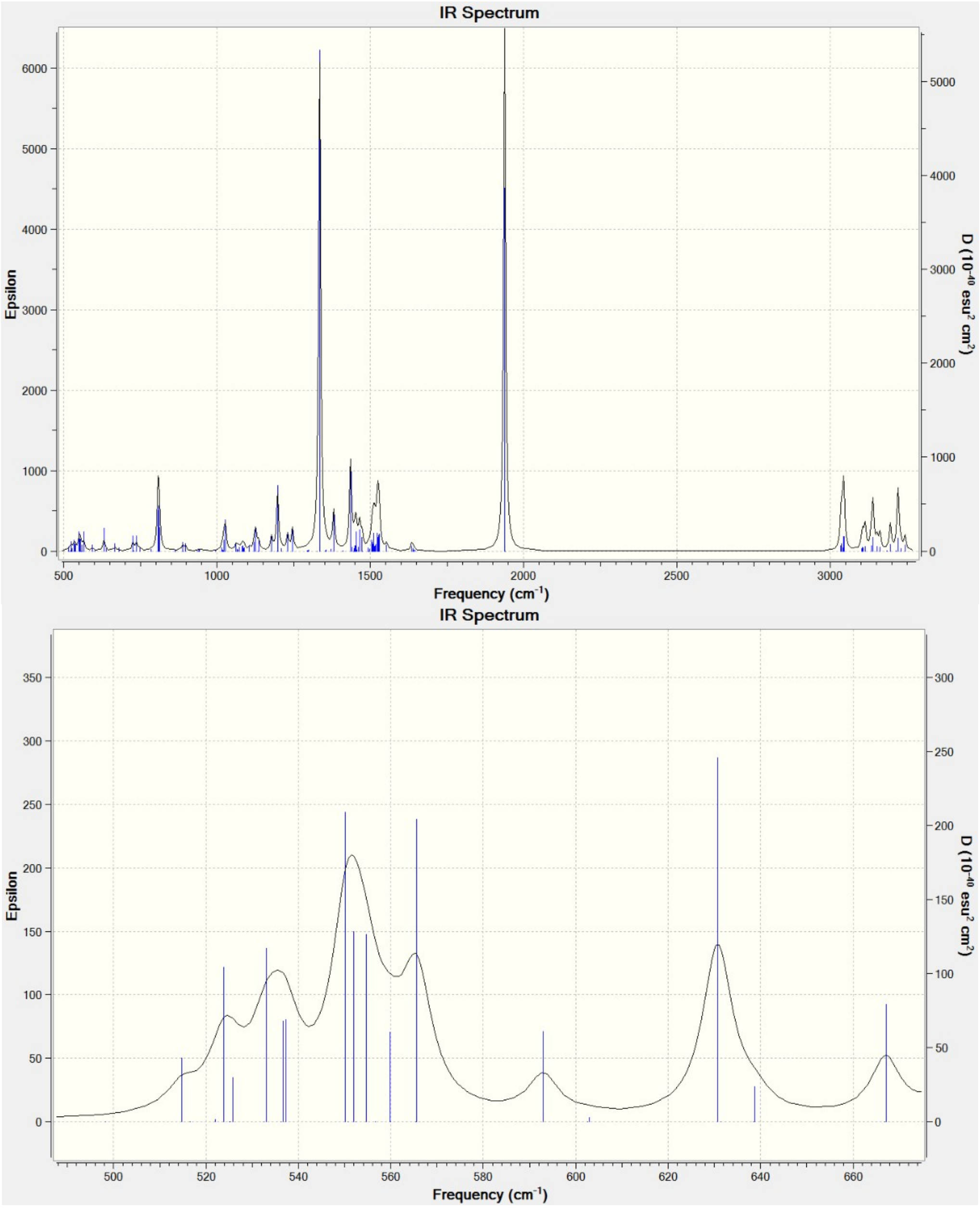

Figure S3: (*top*): The IR spectra of compound (**5**) calculated on an optimized structure by DFT (b3lyp, 6-31G* (C, H, N, O, P) and lanl2dz (Ir). (*bottom*): zoomed in the region of the P=P stretching frequency. The vibration at 498 cm^{-1} is too small to be observed.

The Cartesian Coordinates for 5, 8a and 9

5

C	-3.06329300	3.08838800	-2.33876000
H	-2.89356500	2.38018300	-3.15817300
H	-2.42419700	3.95948500	-2.50619200
H	-4.10899800	3.41799000	-2.41247300
C	-2.76646000	2.45553300	-1.00017300
C	-1.97818600	3.09709100	0.01590100
C	-1.34718600	4.35194300	-0.01217600
H	-1.36351200	4.95172700	-0.91630000
C	-0.69546100	4.83486600	1.14254500
H	-0.20884000	5.80537700	1.11951200
C	-0.67501400	4.07014500	2.32772400
H	-0.18173900	4.45711100	3.21358800
C	-1.29873800	2.81031600	2.35721600
C	-1.46178600	1.90429300	3.46008900
C	-0.99499200	2.21421200	4.86276700
H	-1.84303400	2.36253000	5.54544200
H	-0.38844900	3.12357600	4.87975500
H	-0.39308700	1.39682300	5.27766800
C	-4.34682800	0.66276900	-1.44516500
C	-4.07187600	-0.34693800	-2.40058400
C	-5.14945400	-0.88662900	-3.13629500
H	-4.94533400	-1.66112000	-3.87265200
C	-6.46588000	-0.44609700	-2.92988500
H	-7.28356500	-0.87747100	-3.50233600
C	-6.72163400	0.55372600	-1.97589800
H	-7.74060100	0.89423700	-1.80515400
C	-5.67470600	1.12344600	-1.22400300
C	-5.97891600	2.18769300	-0.18693700
H	-5.54982100	3.16280000	-0.45615700
H	-5.55306400	1.91589800	0.78751100
H	-7.06038900	2.31852800	-0.07197000
C	-2.67014900	-0.86645200	-2.64048000
H	-2.37022000	-1.57450300	-1.85627100
H	-1.92329000	-0.06715100	-2.62537700
H	-2.61085900	-1.38741500	-3.60382700
C	-2.58286100	-0.10720800	4.24186000
C	-1.77744200	-1.19341500	4.66710900

C	-2.26312900	-2.02677700	5.69711700
H	-1.65465200	-2.86689800	6.02467000
C	-3.50926900	-1.78784300	6.29880900
H	-3.86988200	-2.44305100	7.08814000
C	-4.28857300	-0.69881600	5.87407800
H	-5.25552200	-0.50963200	6.33503700
C	-3.84152100	0.15687700	4.84647600
C	-4.70639400	1.31926300	4.39831800
H	-4.20894800	2.28654300	4.55307400
H	-5.65233900	1.33392400	4.95055900
H	-4.92857500	1.24961700	3.32590200
C	-0.42096000	-1.46609900	4.05205300
H	-0.49052900	-1.60381800	2.96615300
H	0.02603100	-2.36763400	4.48658400
H	0.27422300	-0.63195800	4.21765700
C	-3.68056300	-1.05005500	1.28926500
Ir	-2.66754700	0.52954400	1.18374000
N	-3.29029800	1.25089500	-0.63671200
N	-1.91812100	2.34637000	1.19320200
N	-2.14963800	0.76574300	3.16151900
O	-4.34029600	-2.03409100	1.36494600
P	-0.64748400	-0.75307400	0.40990900
P	0.64748400	0.75307400	-0.40990900
C	3.06329300	-3.08838800	2.33876000
H	2.89356500	-2.38018300	3.15817300
H	2.42419700	-3.95948500	2.50619200
H	4.10899800	-3.41799000	2.41247300
C	2.76646000	-2.45553300	1.00017300
C	1.97818600	-3.09709100	-0.01590100
C	1.34718600	-4.35194300	0.01217600
H	1.36351200	-4.95172700	0.91630000
C	0.69546100	-4.83486600	-1.14254500
H	0.20884000	-5.80537700	-1.11951200
C	0.67501400	-4.07014500	-2.32772400
H	0.18173900	-4.45711100	-3.21358800
C	1.29873800	-2.81031600	-2.35721600
C	1.46178600	-1.90429300	-3.46008900
C	0.99499200	-2.21421200	-4.86276700
H	1.84303400	-2.36253000	-5.54544200
H	0.38844900	-3.12357600	-4.87975500
H	0.39308700	-1.39682300	-5.27766800
C	4.34682800	-0.66276900	1.44516500
C	4.07187600	0.34693800	2.40058400
C	5.14945400	0.88662900	3.13629500

H	4.94533400	1.66112000	3.87265200
C	6.46588000	0.44609700	2.92988500
H	7.28356500	0.87747100	3.50233600
C	6.72163400	-0.55372600	1.97589800
H	7.74060100	-0.89423700	1.80515400
C	5.67470600	-1.12344600	1.22400300
C	5.97891600	-2.18769300	0.18693700
H	5.54982100	-3.16280000	0.45615700
H	5.55306400	-1.91589800	-0.78751100
H	7.06038900	-2.31852800	0.07197000
C	2.67014900	0.86645200	2.64048000
H	2.37022000	1.57450300	1.85627100
H	1.92329000	0.06715100	2.62537700
H	2.61085900	1.38741500	3.60382700
C	2.58286100	0.10720800	-4.24186000
C	1.77744200	1.19341500	-4.66710900
C	2.26312900	2.02677700	-5.69711700
H	1.65465200	2.86689800	-6.02467000
C	3.50926900	1.78784300	-6.29880900
H	3.86988200	2.44305100	-7.08814000
C	4.28857300	0.69881600	-5.87407800
H	5.25552200	0.50963200	-6.33503700
C	3.84152100	-0.15687700	-4.84647600
C	4.70639400	-1.31926300	-4.39831800
H	4.20894800	-2.28654300	-4.55307400
H	5.65233900	-1.33392400	-4.95055900
H	4.92857500	-1.24961700	-3.32590200
C	0.42096000	1.46609900	-4.05205300
H	0.49052900	1.60381800	-2.96615300
H	-0.02603100	2.36763400	-4.48658400
H	-0.27422300	0.63195800	-4.21765700
C	3.68056300	1.05005500	-1.28926500
Ir	2.66754700	-0.52954400	-1.18374000
N	3.29029800	-1.25089500	0.63671200
N	1.91812100	-2.34637000	-1.19320200
N	2.14963800	-0.76574300	-3.16151900
O	4.34029600	2.03409100	-1.36494600

8a

79	-1.501906	-0.046870	-0.488601
15	-3.465471	-1.267398	-1.203848
8	-4.316563	-1.309497	1.506599
7	1.361670	0.501678	0.303978
6	0.189935	1.022997	0.046258
6	1.728504	2.828235	0.476445
1	2.150723	3.323679	-0.409006
1	1.888047	3.509218	1.323372
6	2.284272	-3.611099	0.076581

1	2.555498	-4.664537	0.025594
6	-0.604052	2.788215	1.536699
1	-1.589377	2.310601	1.416310
1	-0.123785	2.276891	2.387177
6	2.824881	1.286343	2.177451
1	1.976978	1.430625	2.858748
1	3.596488	2.021054	2.444363
1	3.246518	0.286734	2.345179
6	1.773511	-3.080897	1.252620
1	1.622659	-3.734751	2.111171
6	0.232323	2.514285	0.264037
6	1.419592	-1.733054	1.350895
6	2.442575	1.489742	0.716812
6	0.191751	2.852140	-2.295541
1	1.282178	2.987021	-2.356231
1	-0.251064	3.460115	-3.093789
1	-0.032529	1.800509	-2.525669
6	1.630730	-0.920993	0.220396
6	2.406368	-2.805589	-1.047004
1	2.750499	-3.242430	-1.984345
6	-0.367996	3.257750	-0.939752
1	-1.458077	3.097463	-0.935868
1	-0.207578	4.335933	-0.783486
6	2.071113	-1.451127	-1.007882
6	-0.792409	4.260686	1.871449
1	0.162546	4.802610	1.929567
1	-1.287610	4.367706	2.844265
1	-1.420338	4.770614	1.130445
6	-3.926922	-1.264739	0.396310
6	2.086412	-0.657531	-2.299766
1	1.905528	0.399660	-2.062138
6	0.725108	-1.256489	2.612025
1	0.609151	-0.166631	2.553217
6	3.416695	-0.748390	-3.045304
1	4.270768	-0.484000	-2.407389
1	3.413174	-0.069952	-3.909591
1	3.594538	-1.760709	-3.434106
6	1.501480	-1.579525	3.887839
1	1.520191	-2.660349	4.084808
1	1.017724	-1.105494	4.752821
1	2.543022	-1.233481	3.848808
6	3.688661	1.332642	-0.140087
1	4.139178	0.337687	-0.023236
1	4.430449	2.076950	0.179624
1	3.482999	1.503025	-1.203922
6	0.937682	-1.109086	-3.205591
1	1.059171	-2.159048	-3.507263
1	0.908094	-0.497679	-4.118929
1	-0.036365	-1.019761	-2.700998
6	-0.683899	-1.849677	2.688974
1	-1.272432	-1.610729	1.792286
1	-1.222501	-1.455174	3.561340
1	-0.648932	-2.944433	2.780263

9

15	2.222379	-2.937355	-1.495592
29	1.288285	-1.019146	-0.754936
6	0.585209	0.677472	-0.148130
7	-0.638290	0.889689	0.260208
6	-1.604653	-0.185519	0.342532
6	-2.415538	-0.458619	-0.773739
6	-2.206572	0.191672	-2.126726
1	-1.512333	1.034051	-2.005859
6	-1.533911	-0.801861	-3.077860
1	-0.582820	-1.178043	-2.668890
1	-1.323453	-0.325208	-4.045879
1	-2.179611	-1.672129	-3.263104
6	-3.494937	0.734302	-2.740377
1	-4.191461	-0.073648	-3.005159
1	-3.272092	1.282609	-3.665986
1	-4.022382	1.415713	-2.059058
6	-3.373998	-1.466355	-0.647117
1	-4.016231	-1.702593	-1.495507
6	-3.500221	-2.196656	0.526677
1	-4.255869	-2.976975	0.605551
6	-2.630113	-1.963669	1.582881
1	-2.690282	-2.586480	2.475117
6	-1.653200	-0.968230	1.511631
6	-0.626568	-0.861002	2.622417
1	-0.042500	0.053961	2.465460
6	-1.251581	-0.779823	4.012935
1	-1.996053	0.024630	4.087831
1	-0.473770	-0.595621	4.766516
1	-1.749271	-1.719506	4.290585
6	0.360818	-2.028138	2.542964
1	-0.145729	-2.990717	2.703461
1	1.141047	-1.922488	3.309476
1	0.856877	-2.078355	1.561361

6	-0.962998	2.312173	0.689410
6	0.340903	3.034912	0.310927
1	0.672766	3.686544	1.131242
1	0.170502	3.687014	-0.557111
6	1.385105	1.946097	-0.021300
6	2.157011	2.223697	-1.320755
1	2.636952	3.210877	-1.232849
1	2.971106	1.484991	-1.403621
6	1.311437	2.179126	-2.585587
1	0.889982	1.176795	-2.753122
1	1.914473	2.429252	-3.466874
1	0.475628	2.894023	-2.552767
6	2.368166	1.702677	1.149890
1	1.783360	1.549442	2.072668
1	2.901088	0.754132	0.972439
6	3.380880	2.816023	1.372591
1	3.958159	2.624853	2.285308
1	2.902871	3.799935	1.485612
1	4.098202	2.886297	0.545658
6	-2.168422	2.848286	-0.067431
1	-2.006205	2.854476	-1.152351
1	-2.350376	3.884876	0.247189
1	-3.075654	2.268470	0.151269
6	-1.255850	2.376190	2.183616
1	-2.111656	1.743071	2.454714
1	-1.510810	3.411213	2.448742
1	-0.392413	2.081125	2.792743
6	3.223043	-2.405350	-0.284315
8	3.962001	-2.074157	0.581246

1. Gaussian 09, Revision B.01, Frisch, M. J.; Trucks, G. W.; Schlegel, H. B.; Scuseria, G. E.; Robb, M. A.; Cheeseman, J. R.; Scalmani, G.; Barone, V.; Mennucci, B.; Petersson, G. A.; Nakatsuji, H.; Caricato, M.; Li, X.; Hratchian, H. P.; Izmaylov, A. F.; Bloino, J.; Zheng, G.; Sonnenberg, J. L.; Hada, M.; Ehara, M.; Toyota, K.; Fukuda, R.; Hasegawa, J.; Ishida, M.; Nakajima, T.; Honda, Y.; Kitao, O.; Nakai, H.; Vreven, T.; Montgomery, J. A.; Peralta, Jr., J. E.; Ogliaro, F.; Bearpark, M.; Heyd, J. J.; Brothers, E.; Kudin, K. N.; Staroverov, V. N.; Keith, T.; Kobayashi, R.; Normand, J.; Raghavachari, K.; Rendell, A.; Burant, J. C.; Iyengar, S. S.; Tomasi, J.; Cossi, M.; Rega, N.; Millam, J. M.; Klene, M.; Knox, J. E.; Cross, J. B.; Bakken, V.; Adamo, C.; Jaramillo, J.; Gomperts, R.; Stratmann, R. E.; Yazyev, O.; Austin, A. J.; Cammi, R.; Pomelli, C.; Ochterski, J. W.; Martin, R. L.; Morokuma, K.; Zakrzewski, V. G.; Voth, G. A.; Salvador, P.; Dannenberg, J. J.; Dapprich, S.; Daniels, A. D.; Farkas, O.; Foresman, J. B.; Ortiz, J. V.; Cioslowski, J.; and Fox, D. J.; Gaussian, Inc., Wallingford CT, 2010.
2. Zhao, Y. and Truhlar, D., *Theor. Chem. Acc.*, 2008, **120**, 215-241.
3. (a) Ditchfield, R.; Hehre, W. J.; Pople, J. A. *J. Chem. Phys.* 1971, **54**, 724−728. (b) Hehre, W. J.; Ditchfield, R.; Pople, J. A. *J. Chem. Phys.* 1972, **56**, 2257−2261. (c) Hariharan, P. C.; Pople, J. A. *Theor. Chim. Acta* 1973, **28**, 213−222. (d) Dill, J. D.; Pople, J. A. *J. Chem. Phys.* 1975, **62**, 2921−2923. (e) Francl, M. M.; Pietro, W. J.; Hehre, W. J.; Binkley, J. S.; Gordon, M. S.; DeFrees, D. J.; Pople, J. A. *J. Chem. Phys.* 1982, **77**, 3654−3665.
4. Wadt, W. R.; Hay, P. J. *J. Chem. Phys.* 1985, **82**, 284−298.
5. (a) Höllwarth, A.; Böhme, M.; Dapprich, S.; Ehlers, A. W.; Gobbi, A.; Jonas, V.; Köhler, K. F.; Stegmann, R.; Veldkamp, A.; Frenking, G. *Chem. Phys. Lett.* 1993, **208**, 237−240. (b) Ehlers, A.; Böhme, M.; Dapprich, S.; Gobbi, A.; Höllwarth, A.; Jonas, V.; Köhler, K.; Stegmann, R.; Veldkamp, A.; Frenking, G. *Chem. Phys. Lett.* 1993, **208**, 111−114.
6. (a) Krishan, R.; Binkley, J. S.; Seeger, R.; Pople, J. A. *J. Chem. Phys.* 1980, **72**, 650−654. (b) McLean, A. D.; Chandler, G. S. *J. Chem. Phys.* 1980, **72**, 5639−5648. (c) Clark, T.; Chandrasekhar, J.; Spitznagel, G. W.; Schleyer, P. v. R. *J. Comput. Chem.* 1983, **4**, 294−301.
7. Andrae, D.; Haeussermann, U.; Dolg, M.; Stoll, H.; Preuss, H., *Theoret. chim. Acta* 1990, **77**, 123-141.
